# Drugs inducing interstitial lung disease: in silico analysis of their structures and targets

**DOI:** 10.1186/s10020-026-01559-5

**Published:** 2026-07-21

**Authors:** Mao Tanabe, Shokichi Takahama, Takuya Yamamoto, Ryuichi Sakate

**Affiliations:** Center for Intractable Diseases and ImmunoGenomics (CiDIG), National Institutes of Biomedical Innovation, Health and Nutrition (NIBN), Ibaraki, Osaka 567-0085 Japan

**Keywords:** Drug-induced disease, Interstitial lung disease, ABCG2, Off-target

## Abstract

**Background:**

The molecular mechanisms underlying many drug-induced diseases, namely, the adverse effects caused by medications, remain poorly understood. As we consider that adverse effects result from the unintended targets of a drug, we aimed to clarify the molecular mechanisms of drug-induced diseases based on information regarding off-target interactions.

**Methods:**

First, off-targets were searched in public databases, and four proteins were selected. Subsequently, rare and intractable diseases caused by drugs targeting the proteins were identified through side-effect information. Further, the pharmacological actions of the drugs on the targets and related substructures were investigated using statistical analysis and substructure search.

**Results:**

Of the four proteins, one of the ATP-binding cassette (ABC) transporters, ABCG2, was related to a severe disease, interstitial lung disease (ILD): drugs inducing ILD showed a strong tendency to bind and inhibit ABCG2 and kinases. Moreover, these drugs tended to contain pyrimidine and methoxyethane, both of which are effective at inhibiting ABCG2 and kinases, as substructures. Drugs satisfying both conditions of “binding to ABCG2/kinase” and “having substructure pyrimidine/methoxyethane” were more associated with ILD compared with drugs satisfying only one condition. Among the drugs reported to be associated with a high incidence of ILD, many were strong inhibitors of ABCG2 with or without kinase inhibitory activity; however, some drugs showed little evidence of binding to ABCG2 or kinases. Examples of the latter included folic acid (FA) antagonists, which are known to induce pulmonary fibrosis.

**Conclusions:**

These results suggest that for kinase inhibitors known to induce ILD, inhibition of off-target ABCG2 as well as kinases is likely to be associated with the disease and that many drugs inducing ILD other than known kinase inhibitors also inhibit ABCG2. Some drugs with little evidence of binding to ABCG2 include antifolates, raising the possibility that abnormal FA metabolism is one of the common phenomena of drug-induced ILD, particularly as ABCG2 is a folate efflux transporter related to tissue folate absorption.

**Supplementary Information:**

The online version contains supplementary material available at https://doi.org/10.1186/s10020-026-01559-5.

## Introduction

Drug-induced diseases, the unintentional pharmacological side effects that cause mortality, morbidity and severe symptoms, are serious problems in drug development and clinical drug application. In many cases, the principles for minimizing such undesirable side effects are reduction of dosage and duration of therapy using the drugs (Terada et al. 2015; Longui 2007), as the molecular mechanisms underlying the side effects of the drugs remain unclear (Wei et al. 2021; Sunder et al. 2023). Several researchers suggest that most small-molecule drugs interact with unintended and often unknown biological targets; these off-target interactions may lead to toxicity (Rao et al. 2019; Green et al. 2023). Our previous study using a novel virtual screening method “AI-AAM” also suggested that many “hits” from compound library targeted the same proteins as reference compounds, but were not known to be their targets (Tanabe et al. 2023). Therefore, it is essential for elucidation of molecular mechanisms of side effects to obtain as much information as possible about drug-target interactions.

Drug-target interactions are catalogued in public databases. ChEMBL (Gaulton, et al. 2017; ChEMBL. n.d.) is one of the most well-known databases for quantitative bioactivity interactions and contains experimental data for millions of compounds across thousands of protein targets. However, this database does not provide target coverage for approved drugs. In contrast, DrugBank (Knox et al. 2024) prioritizes approved-drug targets based on relevance and separates drug action targets from drug transporters, drug carriers, and pro-drug conversion enzymes (Knox et al. 2011; DrugBank n.d.). Therefore, targets from the category “Targets” in DrugBank are assumed to confer the desired pharmacological effects, whereas many targets from ChEMBL are non-primary or non-curated targets in DrugBank, which exert unknown or unintended pharmacological effects. To extract the less-curated targets in DrugBank, we first compared the ChEMBL and DrugBank databases and focused on four proteins (ABCG2, ABCB1, MAPT, and TDP1) registered as targets of many drugs in ChEMBL but few drugs in DrugBank. Moreover, we selected six diseases that were identical to or closely related to rare and intractable diseases (RIDs) in Japan based on the side effects assumed to be caused by the binding of drugs to these four proteins. The reason why we selected diseases from the RIDs for analysis was that the definition of diseases for recognition as RIDs in Japan is clear and the RIDs include most of main rare diseases in the world.

Subsequent hierarchical clustering to group similar targets based on their similarity in the combination of ligands showed a strong tendency for ABCG2 to be targeted with kinases, and further statistical analysis clarified that the drugs inducing interstitial lung disease (ILD) had a particularly strong tendency to bind to ABCG2 as well as kinases. Therefore, the present study analyzed data, focusing on drugs that cause ILD.

Drug-induced interstitial lung disease (DI-ILD) is a large and heterogeneous group of adverse drug reactions. More than 500 drugs have been associated with the emergence of DI-ILD, including antineoplastic drugs, antibiotics, antiarrhythmic drugs, and immunosuppressive agents. However, the pathogenetic mechanisms underlying DI-ILD remain largely unknown for most of the drugs (Spagnolo et al. 2022; Bridi et al. 2024; Schwaiblmair et al. 2012). Although we started our analysis by employing data-driven approaches without limiting to a specific disease, this study aimed to clarify the molecular mechanisms of DI-ILD. In this report, we provide an overview of the targets of drugs that induce ILD, the pharmacological action of the drugs on the targets, and the structural features of the drugs acting on the targets.

## Methods

### Construction of compound-target interaction data sets

Compound-target interaction data were extracted from DrugBank (5.1.13_rel250102) and ChEMBL (chembl_35.db) in Janualy 2025 and September 2025, respectively. ChEMBL and/or DrugBank compounds with indications were considered as “drugs,” and among them, only compounds with interactions against at least one protein encoded by a human gene were collected. For the compounds from ChEMBL, we only selected compound–target interactions that were qualified with a confidence_score ≥ 4, assay_type = B (BINDING), target_type = SINGLE PROTEIN and at least one standard-values (IC50, Kd, and Ki) ≤ ‘10,000 nM’ (PCHEMBL ≧ 5), following TargetMine (Chen et al. 2016). Regarding the compounds from DrugBank, only the targets registered in the “Targets” item were extracted.

**Fig. 1:**
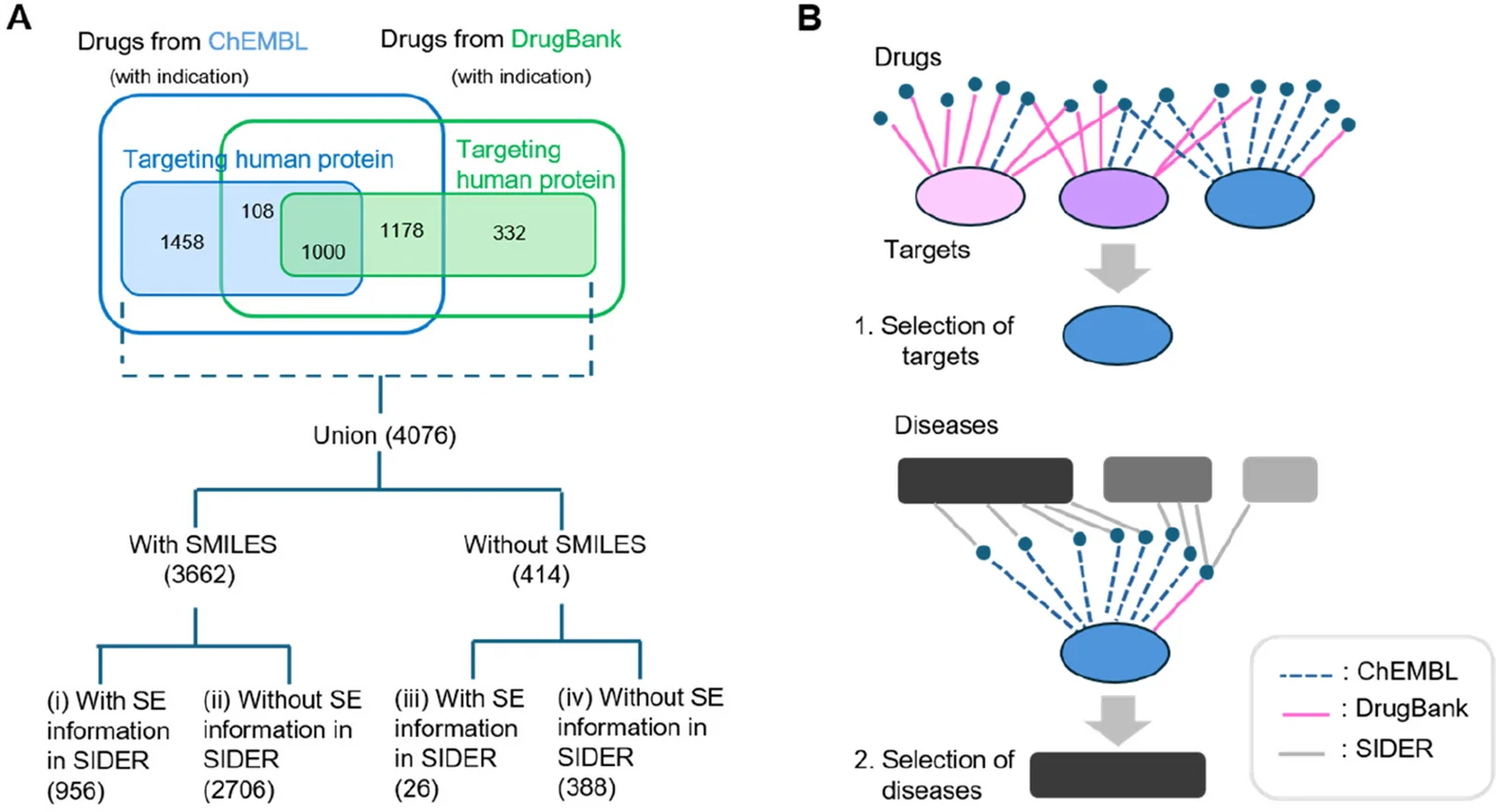
Schematic representation of selection of drugs, target proteins and diseases. **A** Compounds with indication (i.e., drugs) and interactions against at least one human protein were extracted from ChEMBL and DrugBank. The Drug–target interaction data for these drugs were analyzed in this study. Numbers indicate those of drugs. **B** The proteins registered as targets of many drugs in ChEMBL but few drugs in DrugBank were selected as less-curated targets in DrugBank (i.e., ABCG2, ABCB1, TDP1, and MAPT). Then, for each of all diseases registered as side effects in SIDER, the number of drugs causing the disease and binding to one of the proteins was investigated. Among them, the diseases whose responsible drugs had significantly higher ratio to all the other drugs binding to the protein were selected

Subsequently, the search results from ChEMBL (2566 compounds) and DrugBank (2510 compounds) were integrated, and any duplicate compounds were eliminated following the correspondence of DrugBank ID with the ChEMBL ID in the DrugBank database. Consequently, 4076 drugs were retained (Fig. 1). For each drug, the SMILES strings were obtained from the source database (ChEMBL or DrugBank). Data regarding side effects were retrieved using SIDER (SIDER 4.1) (Kuhn et al. 2016; SIDER 4.1: Side Effect Resource n.d.). As the names of the chemicals in SIDER were taken from the STITCH4 database (Kuhn et al. 2014), wherein stereoisomers and salt forms are usually merged into a common identifier, we mapped compounds from ChEMBL and DrugBank to those from SIDER using their names. Of all 982 compounds, the number of compounds from ChEMBL or DrugBank corresponding to multiple drugs from SIDER was 41 (4.18%). For each compound, one SIDER drug was selected referring PubChem. In contrast, the number of compounds from ChEMBL or DrugBank, which corresponded redundantly to the same SIDER drug (i.e., stereoisomers and salt forms) was 70 out of 982 (7.13%). Finally, the integrated compounds from ChEMBL and DrugBank were divided into four groups depending on the presence or absence of SMILES or on the information regarding side effects in SIDER (Fig. 1A i, ii, iii, iv). To compare the results of the substructure search, only compounds with SMILES were analyzed (i.e., i, ii), whereas data were limited to compounds with information regarding side effects (i.e., i, iii) when the compounds causing a side effect and those without side effects were compared. The targets of each compound were proteins registered as targets of the compound in its source database and were limited to proteins encoded by human genes. When a compound was obtained from both ChEMBL and DrugBank, its target genes were integrated. The UniProt IDs of the targets obtained from ChEMBL and DrugBank were made correspond to gene names through KEGG identifiers. Information about the association between UniProt ID and KEGG ID, or gene name and KEGG ID was collected from KEGG version 111.1 in September 2024 using the KEGG API (Application Programming Interface) (KEGG n.d.; Kanehisa et al. 2025). The total number of proteins targeted by at least one compound for analysis was 2718.

### Selection of targets

The proteins registered as targets of many drugs in ChEMBL but few drugs in DrugBank were focused on and regarded as less-curated targets in DrugBank. The ChEMBL proteins targeted by many drugs were ranked according to the total number of drugs targeting them in the database. The total number of drugs in DrugBank was also calculated for each target. Moreover, for each target, the percentages of drugs targeting it from 2566 drugs selected in ChEMBL (A) and that of the drugs targeting it from 2510 drugs selected in DrugBank (B) were calculated and compared by taking the difference between A and B or the ratio of A to B. Considering the results of these calculations, we selected four proteins (ABCG2, ABCB1, TDP1, and MAPT) as less curated targets in DrugBank (Fig. 1B).

### Selection of diseases

For each of the 3960 diseases registered as side effects of drugs in SIDER, the number of drugs causing the disease and binding to ABCG2, ABCB1, TDP1, and MAPT was investigated based on the drug–target interactions registered in ChEMBL and DrugBank, and the percentage of drugs among all drugs binding to the protein (i.e., ABCG2:33, ABCB1:16, MAPT: 32, TDP1:35) was calculated. The diseases were then ranked according to the percentage. Each of disease names from SIDER was roughly made correspond to a KEGG identifier of disease when the name was included in one of the synonyms of the disease corresponding to the KEGG ID, first. Then it was investigated whether the IDs were in the list of RIDs in Japan (KEGG BRITE, jp08407) designated by Ministry of Health, Labour and Welfare (n.d.).

In Japan, “intractable disease” is defined based on specific criteria beyond treatment availability: (1) The pathogenic mechanism remains unclear, (2) No established treatment options are available, (3) The disease is rare, and (4) Long-term medical care is required. Diseases with existing specialized measures—such as cancer, mental illness, and infectious diseases—are not included under this category. Moreover, a specific subset of intractable diseases satisfying additional criteria that the disease can be diagnosed using objective criteria or equivalent standards, with the number of patients in Japan below a certain threshold, are classified as designated intractable diseases (i.e., RIDs) (Ministry of Health, Labour and Welfare: Designated Intractable Diseases.  n.d.).

When a KEGG ID was found in the RID list, the notification number and disease group corresponding to the disease were assigned to it. With the above rough association as clues, the side effects were associated with RIDs in Japan in more detail. The side effects whose responsible drugs targeting each protein were less than three were excluded from the candidate diseases. Then, for each of these candidate diseases, the Chi-square test was used to examine the relationship between two categorical values, one of which was a side effect (drugs inducing the disease/not inducing the disease) and the other was the target (drugs binding to the protein/not binding to the protein). For each disease, when a significantly more responsible drugs compared with other drugs bound to one of the four proteins, the disease was selected (Fig. 1B).

### Hierarchical clustering

Hierarchical clustering, an important and well-established technique in unsupervised machine learning, was performed using the SciPy Library for Python. The Python module scipy.cluster.hierarchy.linkage was used to perform agglomerative clustering, create hierarchical clusters, and visualize the hierarchy of clusters. The input data were in a two-dimensional array regarded as vector data. In the matrix, each row represented the data (target) and each column represented a categorical variable (drug). Each category was represented by a binary variable, where the category was marked as 1 if the drug bound to the target and 0 otherwise. In all, 2718 targets were used for this clustering (see “Construction of compound-target interaction data sets”), and the set of drugs targeting each protein was mapped into a numeric feature vector with length 4076.

### Comparison of the drug group inducing each disease with all drugs

For drug groups inducing toxic epidermal necrolysis (TEN), systemic lupus erythematosus (SLE), ILD, nephrotic syndrome (NS), vasculitis (VAS), or bladder pain (BP) with known ABCG2 substrates, ABCB1 substrates, ABCG2 inhibitors, ABCB1 inhibitors, Ser/Thr kinase inhibitor, and covalent inhibitors added, each drug group was compared with all drugs after excluding the drug group for the following three points.

1. The percentage of drugs whose interactions (i.e., binding) with ABCG2, ABCB1, MAPT, TDP1, serine/threonine (Ser/Thr) kinase, or tyrosine (Tyr) kinase were registered in ChEMBL and/or Drugbank for each drug group
2. The percentage of drugs that were ABCG2 substrates, ABCG2 inhibitors, Ser/Thr kinase inhibitors, Tyr kinase inhibitors, or covalent inhibitors in each drug group
3. The percentage of drugs with ABCG2 inhibitor scaffolds or covalent inhibitor warheads as substructures

The drugs in (2) were investigated by determining whether the drugs were known substrates or inhibitors of ABCG2/ABCB1. We also checked whether the drugs were identical to known covalent inhibitors. Known ABCG2 substrates, ABCB1 substrates, ABCG2 inhibitors, ABCB1 inhibitors, and covalent inhibitors were extracted from literature (Supplementary Table S1) [ABCG2/ABCB1 substrates: (Westover and Li 2015; Sajid et al. 2023; Wang and Fu 2010), ABCG2/ABCB1 inhibitors: (Sajid et al. 2023; Wang and Fu 2010; Bakhsheshian et al. 2013; Stefan 2019; Liu 2019), and covalent inhibitors (Serafim et al. 2023; Vita 2021)]. For each ABCG2 substrate, ABCB1 substrate, ABCG2 inhibitor, and ABCB1 inhibitor, data from multiple studies were integrated, and duplicate compounds were eliminated. Ser/Thr and Tyr kinase inhibitors were obtained from the KEGG BRITE database (br:hsa01001) (Kanehisa et al. 2025). The drugs from ChEMBL and DrugBank were prepared to correspond to these substrates/inhibitors based on their names.

The calculation programs were written in python. For the substructure calculations, python scripts based on RDKit were used. The SMILES query definitions were used to perform the substructure search. These were basic scaffolds of ABC transporter (ABCG2, ABCB1, and ABCC1) inhibitors (Sajid et al. 2023; Namasivayam et al. 2021) (Supplementary Table S2), methoxyethane (Supplementary Table S3), and warheads of covalent inhibitors (Serafim et al. 2023; Vita 2021) (Supplementary Tables S4, S5). The data range was limited to drugs with SMILES information (Fig. 1A; total: 3662), among which 73 substructures were searched.

To identify the relationship between two categorical variables, a chi-square test was performed. One categorical value was side effect [causing the disease (e.g., TEN) *versus* not causing the disease], and the other was one of the followings:

1. Target [targeting the protein (e.g., ABCG2) *versus* not targeting the protein]
2. Pharmacological effect [having an effect (e.g., ABCG2 inhibitor) *versus* not having an effect]
3. Substructure [having the substructure (e.g., pyrimidine) *versus* not having the substructure]

### Principal component analysis (PCA)

Principal component analysis (PCA) was performed to reduce the complexity of the descriptor space and gain an overview of the drugs for similarities and differences. In this study, we implemented PCA using the Scikit-learn Python package. The sets of input data for PCA were matrices with dimensions of 88 × 956, where 88 is the number of variables [i.e., being a substrate/inhibitor (1) or not (0): 7; targeting a protein (1) or not (0): 8; containing a substructure (1) or not (0): 73], and 956 is the number of cases [Supplementary Table S9]. First, the descriptor matrix was standardized using StandardScaler from sklearn.preprocessing. Subsequently, PCA was performed using sklearn.decomposition. Coefficients (loadings) related to the data for each drug yielded two components (PC1 and PC7). Finally, drugs inducing ILD and other diseases were directly compared using PCA to determine group differences. Similarly, Tyr kinase inhibitors, Ser/Thr kinase inhibitors, and covalent inhibitors were compared.

### Relationship between the substructures of drugs and their binding to targets

For drugs that induced ILD, Chi-square test was performed nine times for each target.

When drugs were limited to those binding to or not binding to the target,

1. The independent variable was substructure (with pyrimidine *versus* without pyrimidine), and the dependent variable was side effect (causing ILD *versus* not-causing ILD).
2. The independent variable was substructure (with methoxyethane *versus* without methoxyethane), and the dependent variable was side effect (causing ILD *versus* not-causing ILD).
3. The independent variable was substructure (with both pyrimidine and methoxyethane *versus* others), and the dependent variable was side effect (causing ILD *versus* not-causing ILD).

When drugs were limited to those with a specific substructure (i.e., pyrimidine, methoxyethane, or both), the independent variable was the target (binding to the target *versus* not-binding to the target) and the dependent variable was ILD (causing ILD *versus* not-causing ILD).

### Integration of literature information with the results of this study (Drugs inducing ILD only)

To the results of this study, information on drugs associated with a high incidence of ILD (Jiang et al. 2004; Skeoch et al. 2018) and those that strongly inhibit ABCG2 (Deng et al. 2023) was integrated. The proportion of strong ABCG2-inhibitors to two of the three drug groups [i.e., drugs with a high incidence of ILD, drug inducing ILD (not high rate), drugs not inducing ILD] were compared, when the drugs were limited to kinase inhibitors or other drugs. Drugs that reduced the relative transport activity of ABCG2 to less than 25% of the vehicle control were categorized as strong inhibitors following the above literature (Deng et al. 2023).

### Statistical analysis

Statistical analysis was performed using Microsoft Excel with statistical analysis software (BellCurve for Excel, Ver. 4.09). The relationship between two categorical variables was analyzed using the Chi-square test (two-tailed). When the Chi-square test was significant, the Phi (ϕ) coefficient was calculated to quantify the strength of association between categorical variables. To test whether there is a difference between two samples (groups), Mann–Whitney U tests were used. All these tests were 2-tailed and *p* < 0.05 significant. For multiple hypothesis tests in Chi-square tests or Mann–Whitney U tests, Holm correction was applied after the *p*-values were obtained. To analyze difference in the proportions for two independent samples, z-Test was performed. This test was one-tailed and *p* < 0.05 significant.

## Results

### Selection of targets

It is conceivable that many drug-target interactions registered in ChEMBL are less curated in DrugBank. The results of the comparison between the top ten proteins registered as targets of many drugs in ChEMBL and DrugBank confirmed that a marked difference between the two (Supplementary Table S6). To extract the less-curated targets in DrugBank that are thought to be implicated in side effects, the percentage of drugs targeting these to all drugs in ChEMBL (A) and that of the drugs targeting these to all drugs in DrugBank (B) were compared (Table 1). Although the protein group included many kinases, these were excluded from the candidates at present because kinases and aminergic GPCRs are known to exhibit the greatest intra- and inter-gene family promiscuity (Paolini et al. 2006) and are not always regarded as unintended targets. First, three proteins, ABCG2 (ABC transporters), MAPT (microtubule-associated protein tau), and TDP1 (tyrosyl-DNA phosphodiesterase 1), which had higher scores in both differences between A and B, and the ratio of A to B, were selected. As for KCNH2 and HDAC6, the A-to-B ratios were not particularly high and these were excluded. Because we found many dual ABCB1/ABCG2 inhibitors (SIDER 4.1 : Side Effect Resource n.d.; Stefan et al. 2022), ABCB1 was also added to the three proteins, although all of the percentage of drugs targeting the protein in ChEMBL and DrugBank (A: 1.169, B: 0.359), difference between them (A-B = 0.81), and the ratio of them (A/B = 3.26) were smaller than the other three proteins (i.e., ABCG2, MAPT, and TDP1).

**Table 1:** Examples of proteins as targets of many drugs in ChEMBL but few drugs in DrugBank

UniProt_ID	Gene name	ChEMBL (%)	DrugBank (%)		
		A	B	A-B	A/B
Q12809	KCNH2	5.77	1.55	4.21	3.71
Q9NUW8	**TDP1**	4.05	0.00	4.05	
P10636	**MAPT**	3.98	0.08	3.90	49.89
P07949	RET	4.21	0.48	3.73	8.80
P36888	FLT3	4.33	0.72	3.61	6.03
P00519	ABL1	3.31	0.52	2.79	6.40
P35968	KDR	3.66	1.04	2.63	3.54
P00533	EGFR	3.74	1.16	2.59	3.24
P06239	LCK	2.73	0.20	2.53	13.69
O14976	GAK	2.49	0.04	2.45	62.60
O94804	STK10	2.42	0.04	2.38	60.65
Q9UBN7	HDAC6	2.49	0.12	2.37	20.87
Q08345	DDR1	2.46	0.12	2.34	20.54
P09619	PDGFRB	3.08	0.76	2.32	4.07
Q96GD4	AURKB	2.38	0.16	2.22	14.92
P07948	LYN	2.34	0.20	2.14	11.74
O60674	JAK2	2.65	0.56	2.09	4.75
P11362	FGFR1	2.69	0.60	2.09	4.50
P08581	MET	2.46	0.40	2.06	6.16
Q9UNQ0	**ABCG2**	2.07	0.04	2.03	51.84
P12931	SRC	2.26	0.24	2.02	9.46
Q06187	BTK	2.34	0.32	2.02	7.34
Q9H2G2	SLK	2.07	0.08	1.99	25.92
P52333	JAK3	2.30	0.40	1.90	5.77

“A” indicates the percentage of drugs targeting each protein to all selected 2566 drugs in ChEMBL, and “B” indicates that of the drugs targeting it to all selected 2510 drugs in DrugBank. Proteins are arranged in the order of “A–B.” Gene names in bold letters indicate the proteins finally selected as less curated targets in DrugBank

### Selection of diseases

After selecting targets inferred to be involved in the pathogenesis of drug-induced diseases, the specific diseases caused by these targets and those related to RIDs in Japan were investigated. These results are presented in Supplementary Tables S7 and S8. There were six RIDs with at least three responsible drugs targeting ABCG2. Among them, as both Stevens-Johnson syndrome and TEN in SIDER or RIDs in Japan are considered to have the same pathophysiology, only the latter was selected for analysis. Significantly more drugs that induced ILD bound to ABCG2 than other drugs (*p* < 0.01). A similar tendency was also observed for TEN, BP, and NS (*p* < 0.01). The maximum Phi (ϕ) coefficient was 0.242, which was the value of ILD, and the others ranged from 0.092 to 0.101. For ABCB1, significantly more drugs inducing ILD targeted the protein (*p* < 0.05, ϕ = 0.074). Regarding MAPT, drugs binding to the protein and other drugs were not independent in relation to the causability of any disease. As for TDP1, significantly more drugs inducing SLE and VAS bound to the protein than the other drugs (SLE: *p* < 0.01, ϕ = 0.164; VAS: *p* < 0.05, ϕ = 0.076). Hence, TEN, SLE, ILD, NS, VAS, and BP were inferred to be induced through “unknown” targets (i.e., ABCG2, ABCB1, MAPT, and TDP1) and were selected for further analysis.

### Grouping of targets based on their similarity in ligands

**Fig. 2:**
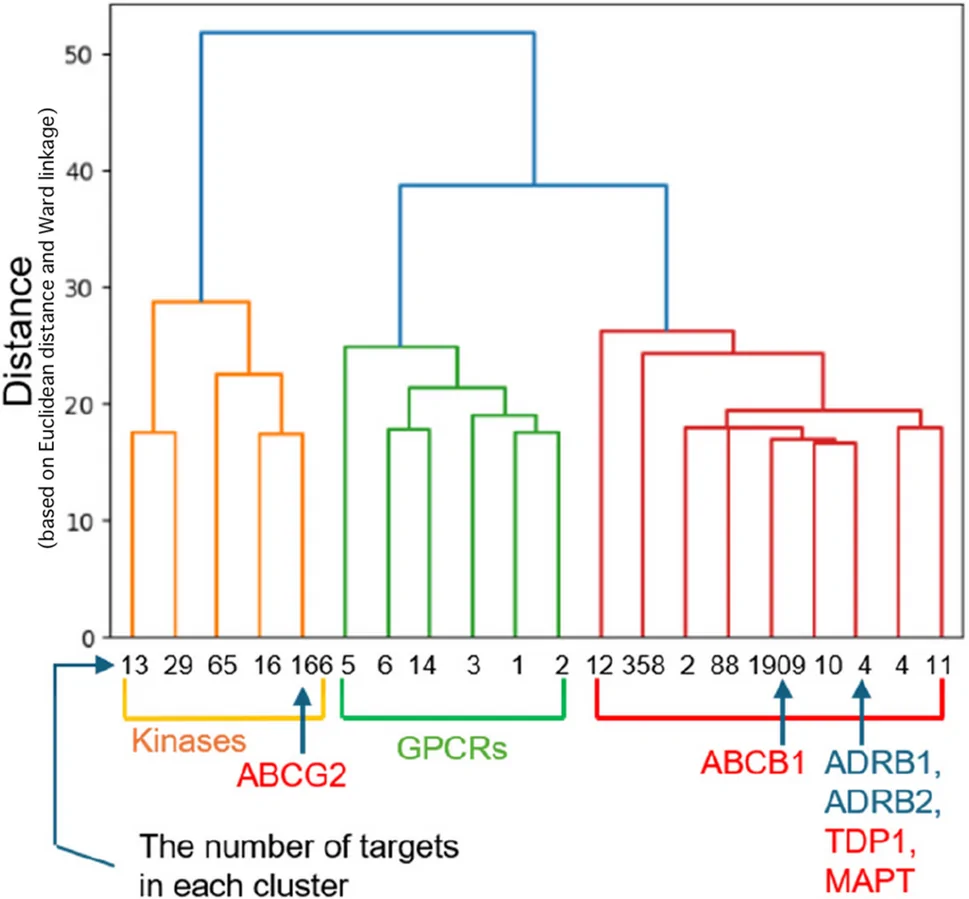
Hierarchical clustering of targets by ligands. Proteins are clustered based on the drugs targeting them. The data elements are stored at the leaves of the binary merge tree. The vertical axis represents the distance between clusters calculated using the Euclidean distance and the Ward linkage method. Groups highlighted with orange and green mainly include kinases and GPCRs, respectively. ABCG2 is included in one of orange groups and TDP1 and MAPT, together with ADRB1 and ADRB2, are included in one of the other groups (red)

To search for proteins targeted by a drug together with the above-mentioned less-curated targets in DrugBank, hierarchical clustering was used, in which similar targets were grouped together based on their similarity in the combination of ligands. The results for all targets, ordered by hierarchical clustering, are displayed in Fig. 2.

The results indicated that the targets in each cluster had characteristics in functional categories. One of the three major clusters (orange) mainly included kinases, whereas another cluster (green) contained mostly GPCRs. The cluster including ABCG2 belonged to a major cluster of kinases. Thus, ABCG2, which was selected as a less-curated target in DrugBank in the previous section, was also included as a different type of protein in the kinase group by hierarchical clustering. Drugs that target kinases may bind to ABCG2. Similarly, TDP1 and MAPT were included in a cluster together with different types of proteins (i.e., adrenergic receptors) ADRB1 and ADRB2. These are also likely to be targeted by drugs that target ADRB1/2.

### Comparison of the drug group inducing each disease with all drugs

As shown in Supplementary Table S7, of the four proteins (ABCG2, ABCB1, MAPT, and TDP1), ABCG2 is especially targeted by several drugs that induce severe diseases. Moreover, the hierarchical clustering of targets suggests that the protein is targeted by a drug together with kinases (Fig. 2). Several studies have recently addressed that most, but not all Tyr kinase inhibitors can inhibit the functions of ABC transporters by binding to their ATP-binding sites (Wang and Fu 2010).

Although ChEMBL accumulates massive amounts of data on quantitative bioactivity interactions, including experimental data on millions of compounds across thousands of protein targets, it does not cover all combinations of drugs and targets. Therefore, absence of data on the relationship between a compound and its target does not always mean that the compound does not bind to the target. Moreover, to know not only the affinity of a drug to a target, but also the pharmacological action, investigation from a viewpoint other than the information of drug-target interactions in the databases is needed. For this purpose, it was examined whether the drugs were identical with known ABCG2/ABCB1 substrates, inhibitors, Ser/Thr kinase inhibitors, and Tyr kinase inhibitors.

Additionally, a substructure search was performed using the basic scaffolds of ABCG2 inhibitors (Supplementary Table S2), many of which were pyrimidine itself or contained pyrimidine, which is also known to prevent ATP from binding and inhibit kinase activity by mimicking the adenine part of the ATP molecule (Namasivayam et al. 2021; Baillache and Unciti-Broceta 2020; Jug and Ilas 2025). Moreover, many drugs that induce ILD contain methoxyethane as a substructure.

The methoxy moiety is not known to be a basic scaffold of ABCG2, but is one of the substructures of the ABCG2 inhibitor (Namasivayam et al. 2021, 2022) and is reported to be effective for inhibiting ABCG2 or other ABC transporters (Valdameri et al. 2012; Zanden et al. 2005). Furthermore, it favors EGFR inhibition (Șandor et al. 2023). We also found many drugs known as covalent inhibitors among the drugs inducing ILD and assumed that drugs inducing such severe side effects not only bound to their targets but also strongly inhibited them. To evaluate this hypothesis, methoxyethane and the warheads of covalent inhibitors were added to the queries in a substructure search.

Considering the above, for drug groups inducing TEN, SLE, ILD, NS, VAS, or BP with known ABCG2 substrates, ABCB1 substrates, ABCG2 inhibitors, ABCB1 inhibitors, Ser/Thr kinase inhibitors, Tyr kinase inhibitors, and covalent inhibitors, a comparison of each drug group with all drugs after excluding the drug group was performed based on the following three points (1–3).

**Fig. 3:**
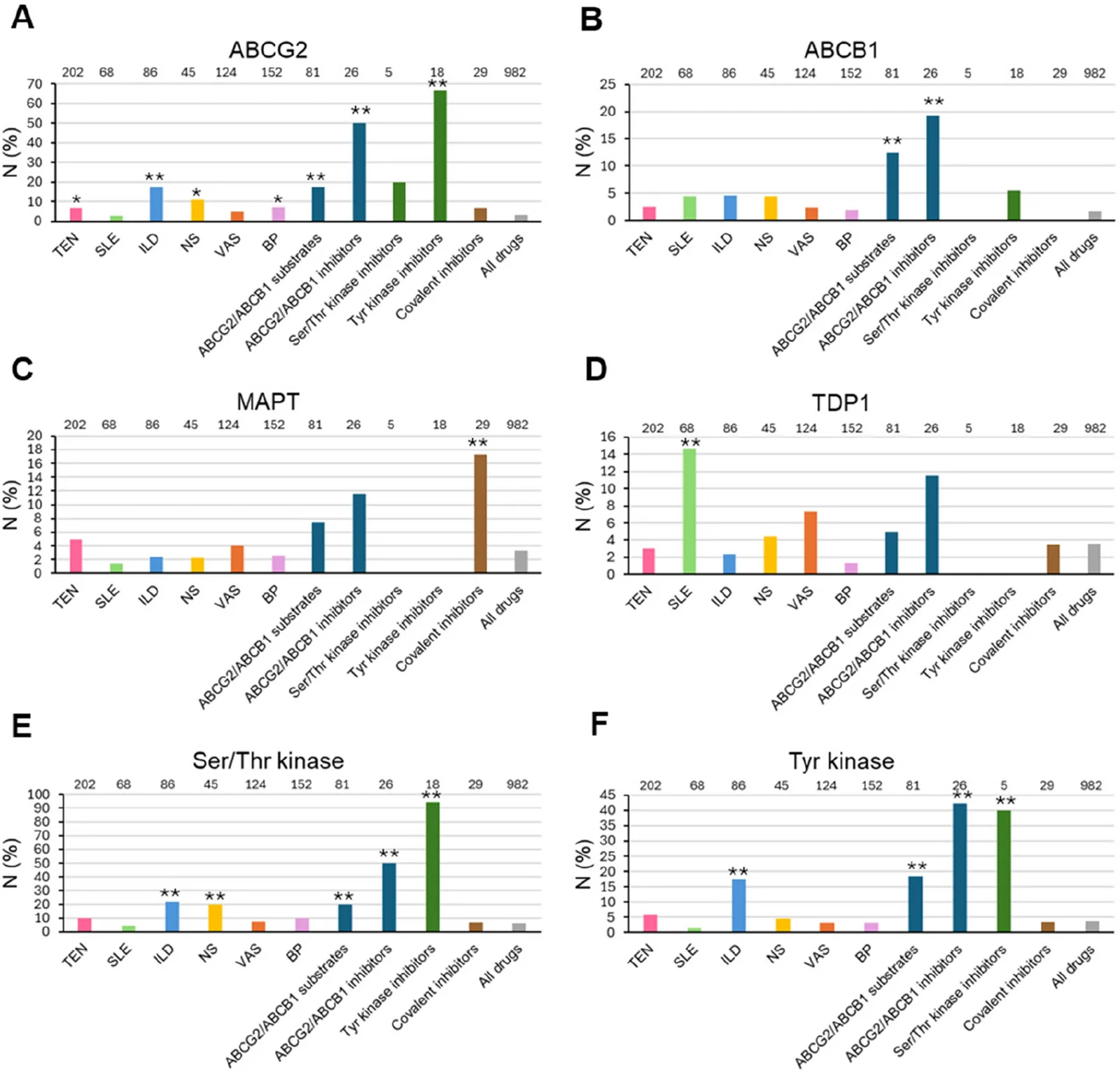
Distribution of the percentage of drugs targeting a protein to each of the drug groups. Each protein is shown as gene name: ABCG2 (**A**), ABCB1(**B**), MAPT (**C**), TDP1 (**D**), Ser/Thr kinase (**E**), or Tyr kinase (**F**). The upper number of each bar is the total number of drugs. Chi-Square tests with Holm correction for multiple comparisons were done to calculate *p* values [the number of multiple comparisons is 11 (**A**, **B**, **C**, **D**) or 10 (**E**, **F**); * p < 0.05, ** p < 0.01]

1. The percentage of drugs whose binding to ABCG2, ABCB1, MAPT, TDP1, Ser/Thr kinase, or Tyr kinase was registered in ChEMBL and/or DrugBank for each of the drug groups (Fig. 3, Supplementary Fig. S1)

The results, shown in Fig. 3A-D, indicate that, among the drugs inducing TEN, ILD, NS or BP, more drugs tended to bind to ABCG2 (TEN: *p* < 0.05, ϕ = 0.101; ILD: *p* < 0.01, ϕ = 0.242; NS: *p* < 0.05, ϕ = 0.094, BP: *p* < 0.05, ϕ = 0.092); among the drugs inducing SLE, more drugs tended to bind to TDP1 (SLE: *p* < 0.01, ϕ = 0.164). These results have already been used for the selection of diseases (Supplementary Table S7). Newly yielded results indicate that among the ABCG2/ABCB1 substrates, more drugs tended to bind to ABCG2 (*p* < 0.01, ϕ = 0.232), ABCB1 (*p* < 0.01, ϕ = 0.254), Ser/Thr kinase (*p* < 0.01, ϕ = 0.166), and Tyr kinase (*p* < 0.01, ϕ = 0.232). Similarly, among ABCG2/ABCB1 inhibitors, more drugs tended to bind to all of ABCG2 (*p* < 0.01, ϕ = 0.427), ABCB1 (*p* < 0.01, ϕ = 0.229), Ser/Thr kinase (*p* < 0.01, ϕ = 0.296), and Tyr kinase (*p* < 0.01, ϕ = 0.334) compared to other drugs. Moreover, Fig. 3A shows that Tyr kinase inhibitors included more drugs that bound to ABCG2 than other drugs (*p* < 0.01, ϕ = 0.480). In contrast, the results shown in Fig. 3E and F indicate that, among drugs inducing ILD, ABCG2/ABCB1 substrates, ABCG2/ABCB1 inhibitors, and kinase inhibitors, more drugs tended to bind to Ser/Thr kinases (all: *p* < 0.01, ILD: ϕ = 0.296, ABCG2/ABCB1 substrates: ϕ = 0.166, ABCG2/ABCB1 inhibitors: ϕ = 0.296) and Tyr kinases (all: *p* < 0.01, ILD: ϕ = 0.222, ABCG2/ABCB1 substrates: ϕ = 0.232, ABCG2/ABCB1 inhibitors: ϕ = 0.334). The tendency to bind to Ser/Thr kinases was also seen among the drugs inducing NS (NS: *p* < 0.01, ϕ = 0.123).

**Fig. 4:**
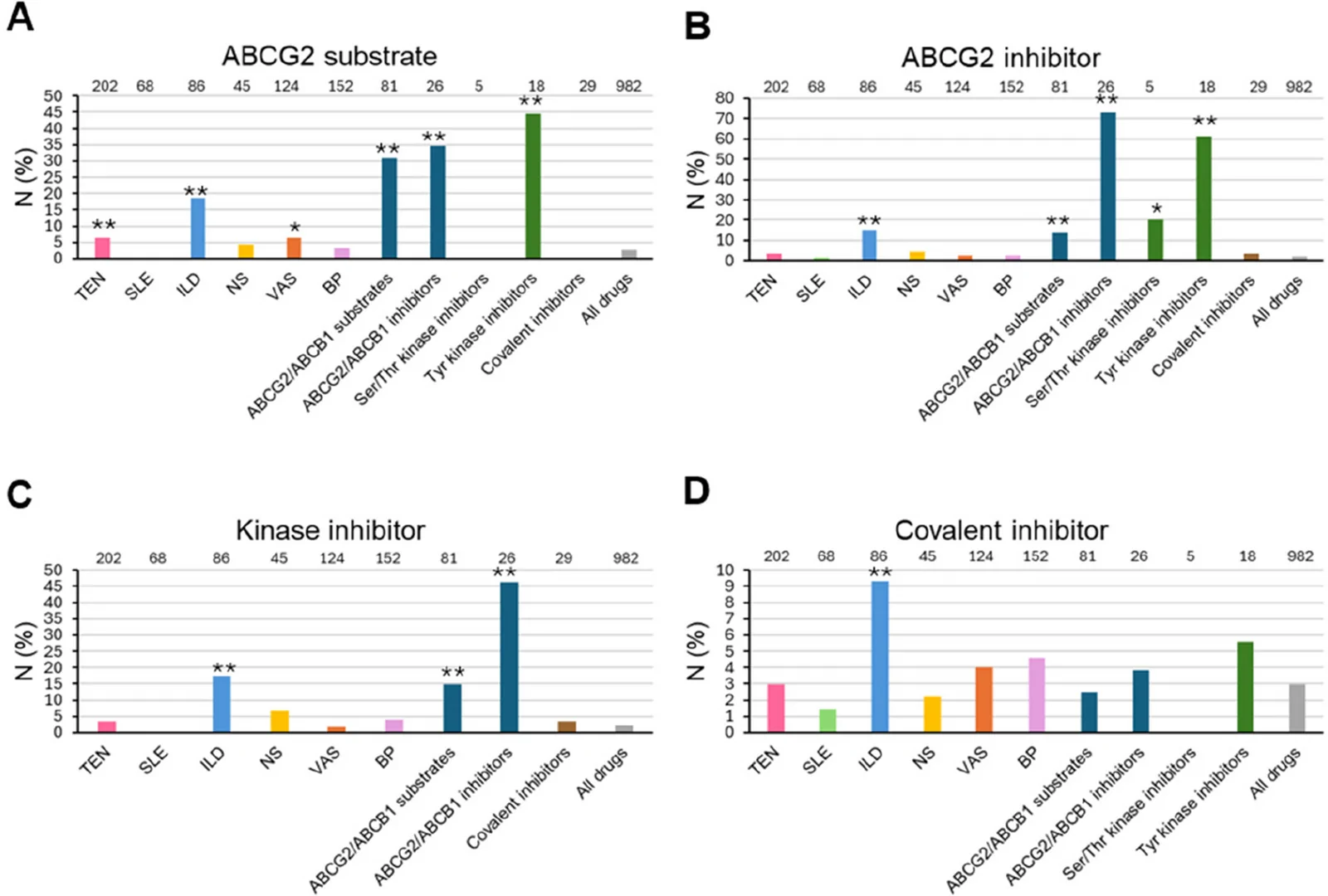
Distribution of the percentage of drugs of a category to each of the drug groups. The categories are ABCG2 substrate (**A**), ABCG2 inhibitor (**B**), (Ser/Thr or Tyr) kinase inhibitor (**C**), and covalent inhibitor (**D**). The upper number of each bar is the total number of drugs. Chi-Square tests with Holm correction for multiple comparisons were done to calculate *p* values [the number of multiple comparisons is 11 (A, B), 9 (C) or 10 (D); * p < 0.05, ** p < 0.01]

2) The percentages of drugs that were ABCG2 substrates, ABCG2 inhibitors, Tyr kinase inhibitors, Ser/Thr inhibitors, or covalent inhibitors in each of the drug groups (Fig. 4, Supplementary Fig. S2)

The results, shown in Fig. 4A, indicate that among the drugs inducing TEN, ILD, or VAS, ABCG2/ABCB1 substrates, ABCG2/ABCB1 inhibitors, and Tyr kinase inhibitors, more drugs tended to be identical to ABCG2 substrates than to other drugs (TEN: *p* < 0.01, ϕ = 0.126; ILD: *p* < 0.01, ϕ = 0.315; VAS: *p* < 0.05, ϕ = 0.094; ABCG2/ABCB1 substrates: *p* < 0.01, ϕ = 0.539, ABCG2/ABCB1 inhibitors: *p* < 0.01, ϕ = 0.336; Tyr kinase inhibitors: *p* < 0.01, ϕ = 0.363). Figure 4B shows that drugs inducing ILD, ABCG2/ABCB1 substrates, ABCG2/ABCB1 inhibitors, Ser/Thr kinase inhibitors, and Tyr kinase inhibitors tended to include ABCG2 inhibitors at higher rates than other drugs (ILD: *p* < 0.01, ϕ = 0.296; ABCG2/ABCB1 substrates: *p* < 0.01, ϕ = 0.253; ABCG2/ABCB1 inhibitors: *p* < 0.01, ϕ = 0.851; Ser/Thr kinase inhibitors: *p* < 0.05, ϕ 0.094, and Tyr kinase inhibitors: *p* < 0.01, ϕ = 0.587). As shown in Fig. 4C, drugs inducing ILD, ABCG2/ABCB1 substrates and ABCG2/ABCB1 inhibitors tended to include kinase inhibitors at higher rates than other drugs (all *p* < 0.01, ILD: ϕ = 0.309; ABCG2/ABCB1 substrates: ϕ = 0.253; ABCG2/ABCB1 inhibitors: ϕ = 0.852). The results, shown in Fig. 4D, indicate that among the drugs inducing ILD, more drugs tended to be identical to covalent inhibitors than to other drugs (*p* < 0.01, ϕ = 0.116).

**Fig. 5:**
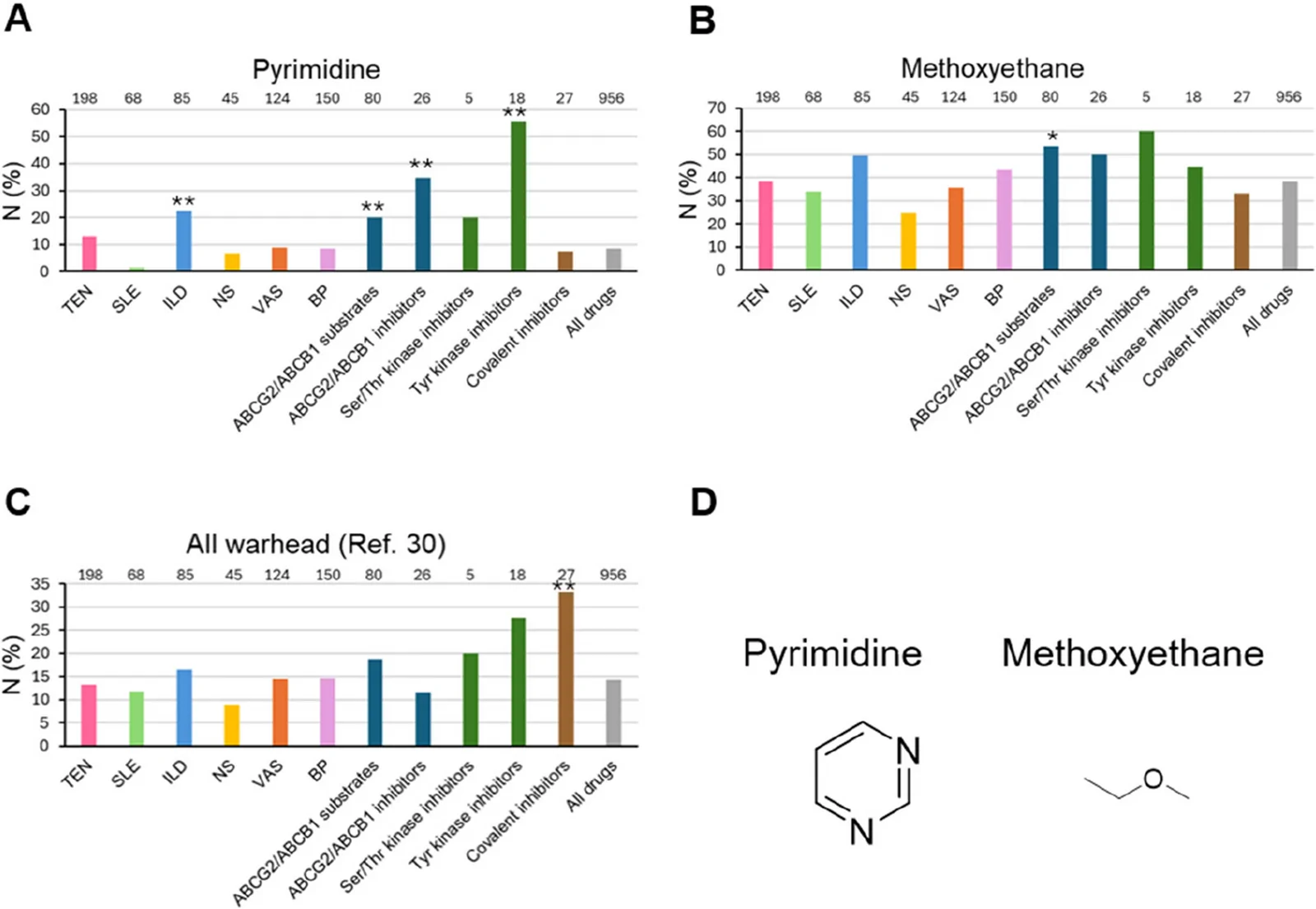
Distribution of the percentage of drugs containing a substructure to each of the drug groups. The substructures are pyrimidine (**A**), methoxyethane (**B**), or warheads of covalent inhibitor (**C**). The upper number of each bar is the total number of drugs. Chi-Square tests with Holm correction for multiple comparisons were done to calculate *p* values [the number of multiple comparisons is 11; * p < 0.05, ** p < 0.01]. **D** shows the structures of pyrimidine and methoxyethane

3) The distribution of the percentage of drugs containing pyrimidine, methoxyethane, ABCG2 inhibitor scaffolds, or warheads of covalent inhibitors as substructures for each drug group (Fig. 5, Supplementary Fig. S3)

The results, shown in Fig. 5A, indicate that among the drugs inducing ILD, ABCG2/ABCB1 substrates, ABCG2/ABCB1 inhibitors, and Tyr kinase inhibitors, more drugs tend to contain pyrimidine as a substructure than other drugs (all: *p* < 0.01, ILD: ϕ = 0.156, ABCG2/ABCB1 substrates: ϕ = 0.125, ABCG2/ABCB1 inhibitors: ϕ = 0.157, Tyr kinase inhibitors: ϕ = 0.234). In contrast, Fig. 5B shows that ABCG2/ABCB1 substrates tended to contain methoxyethane at higher rates than the other drugs (*p* < 0.05, ϕ = 0.095). Figure 5C indicates that among the drug group of covalent inhibitors, more drugs tended to contain warheads as substructures than other drugs (*p* < 0.01, ϕ = 0.093).

The distribution of the percentage of drugs containing ABCG2 inhibitor scaffolds was almost the same as that of pyrimidine only and is omitted from this Figure (Supplementary Fig. S3).

Collectively, these data indicate that drugs inducing ILD, among the selected six diseases, are tightly related to some of the targets, pharmacological effects, substructures investigated. Then, to investigate the features of drugs inducing ILD with respect to targets, pharmacological effects, substructures, and their relationships, PCA was performed. Although individual features are shown in the “Comparison of drug group inducing each disease with all drugs” section, the purpose of this analysis was to integrate the features and group the drugs based on them.

### PCA

**Fig. 6:**
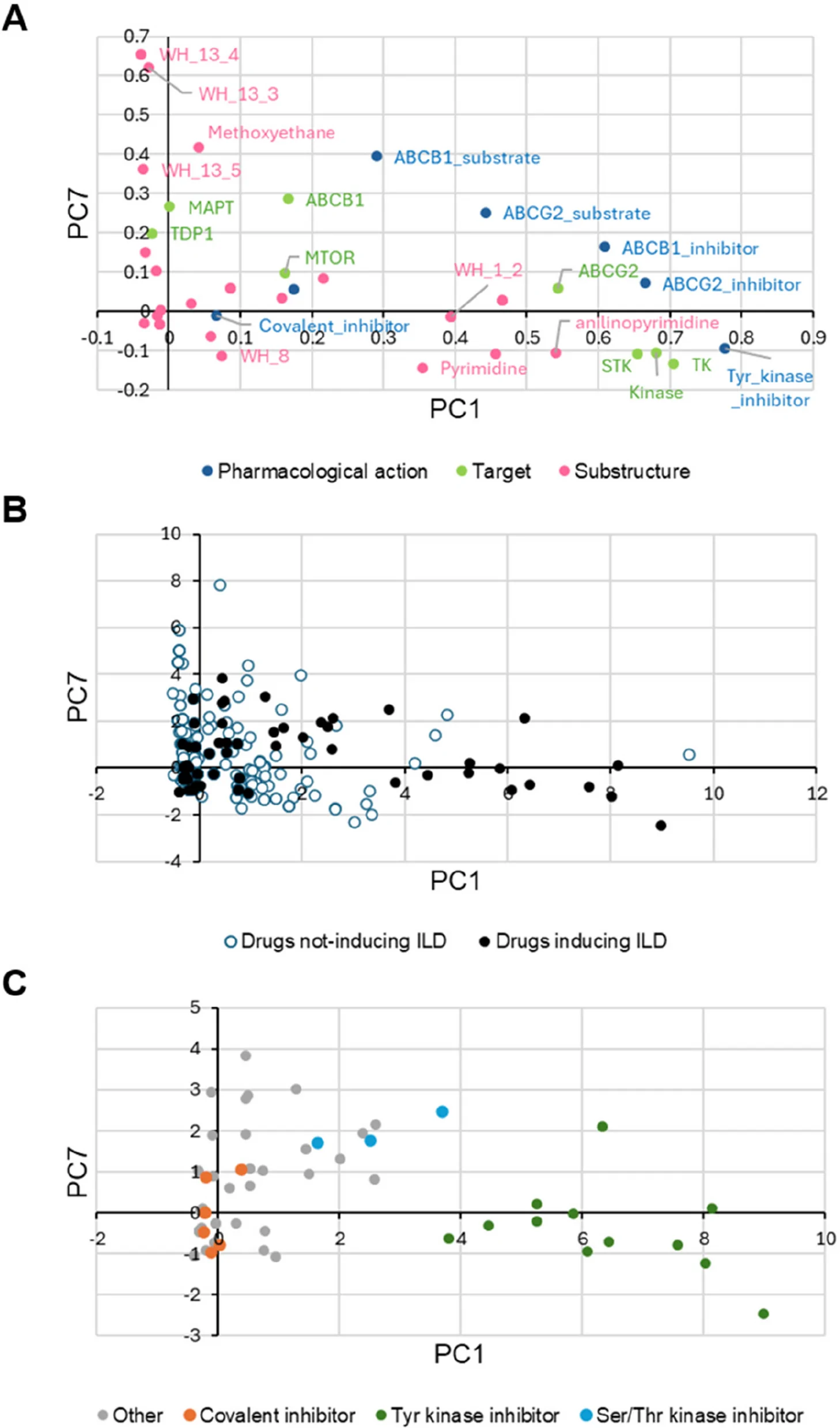
PCA loading plot and score plot of the molecular descriptors for drugs. PCA loading plot (**A**) represents the PCA biplot of drugs with characteristics (targets, pharmacological effects, and substructures) as statistical valuables. In the plot, pharmacological actions are marked as blue dots, targeting proteins are marked as green, and substructures are marked as pink dots. Score plots (**B**, **C**) show biplots of the most informative PCs (i.e., PC1 and PC7) in this model and represent the distribution of PCA component weights across the group (**B** the drugs inducing ILD and the other drugs, **C** Tyr kinase inhibitors, Ser/Thr kinase inhibitors, and covalent inhibitors). Statistical tests were performed using Mann–Whitney U tests (**B**) and Mann–Whitney U tests with Holm correction (**C** the number of multiple comparisons was 3) (see text)

The PCA loading plot (Fig. 6A) visualizes the principal components derived from the original variables**.** In the score plot (Fig. 6B, C), the variables (drugs) are represented by points colored according to their attributes. In score plot B, only PC1 of the two principal components (PC1 and PC7) differed between the drugs that induced ILD and the other drugs (*p* < 0.001), with the drugs that induced ILD having higher component weights. Score plot C showed a clear separation in PC1 among Tyr kinase inhibitors, Ser/Thr kinase inhibitors, and covalent inhibitors; both Tyr kinase inhibitors and Ser/Thr kinase inhibitors had higher component weights than that of covalent inhibitors (*p* < 0.001 and *p* < 0.05, respectively), and the component weights were higher for Tyr kinase inhibitors than for Ser/Thr kinase inhibitors (*p* < 0.01). In contrast, the PC7 component weights were higher in the Ser/Thr kinase inhibitors than in the Tyr kinase inhibitors (*p* < 0.05) and covalent inhibitors (*p* = 0.05). The loadings of the variables in the plot (Fig. 6A) show that the separation along PC1 is described by differences in certain characteristics (i.e., targets, pharmacological effects, and substructures). In this case, PC1 alone described 11.5% variation, which was strongly correlated to being Tyr kinase inhibitors (correlation: 0.777) and ABCG2/ABCB1 inhibitors (0.609, 0.666), binding to Ser/Thr kinases (0.654), Tyr kinases (0.705), and ABCG2 (0.543), and contained the substructures known as ABCG2 inhibitor scaffolds (i.e., anilinopyrimidine: 0.540, quinazoline: 0.457, pyrimidine: 0.354). In contrast, the PC7 share of 4.1% in the total variation was mainly attributed to two of the substructures known as Ser/Thr warheads of covalent inhibitors (0.620, 0.655) and methoxyethane (0.418), all of which contained the methoxy moiety.

### Relationship between the substructures of a drug and its binding to targets

**Fig. 7:**
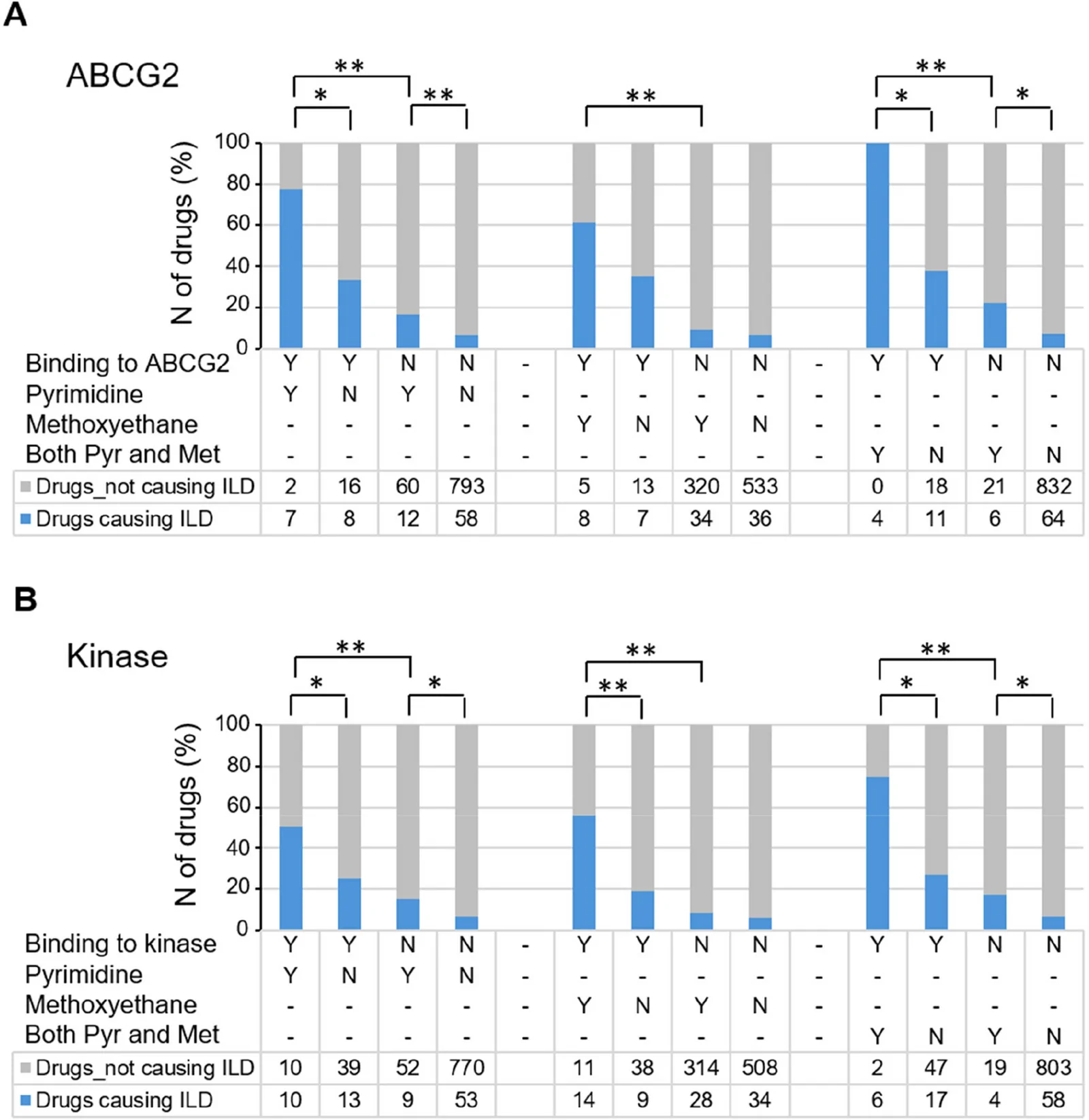
Percentage distribution of drugs inducing ILD. **A** In total, 33 drugs bound to ABCG2, in which 15 drugs caused ILD whereas 18 did not. Among the 15 drugs causing ILD, the substructures pyrimidine, methoxyethane, and both occurred 7, 8, and 4 times, respectively. The distribution of ILD induction differed for the presence of substructures (pyrimidine or both of pyrimidine and methoxyethane) and their absence (*p* < 0.05). **B** For the total 72 drugs binding to at least one kinase, 23 drugs induced ILD, among which 10, 14, and 6 drugs contained pyrimidines, methoxyethanes, and both, respectively. The distribution of ILD induction differs for the presence of substructures (pyrimidine, methoxyethane or both of them) and their absence (pyrimidine: *p* < 0.05, methoxyethane: *p* < 0.01, both pyrimidine and methoxyethane: *p* < 0.05). In the figure, “Y” indicates “bind to protein(s)” or “contain substructure(s)”, while “N” indicates “not bind to protein(s)” or “not contain substructure(s)”. Chi-Square tests with Holm correction for multiple comparisons were done to calculate *p* values (the number of multiple comparisons is 3 in each of left, middle and right graphs of A and B; * p < 0.05, ** p < 0.01)

As shown in Fig. 3A and B, there were much more drugs targeting ABCG2 than those targeting ABCB1, and ABCG2 was more strongly associated with ILD than ABCB1, ABCG2, with kinases, was selected as target for analysis. The results are presented in Fig. 7. When the drugs were limited to those binding to ABCG2 (Fig. 7A), significantly more ILD-causing cases were found among the drugs with pyrimidine with significantly fewer cases among the drugs without pyrimidine (*p* < 0.05, ϕ = 0.398, 7A left graph). Although drugs with methoxyethane and drugs without methoxyethane were not independent of the cause of ILD when the drugs were limited to those binding to ABCG2, there were significantly more cases of ILD caused among the drugs binding to ABCG2 and significantly fewer cases among the drugs not binding to it when the drugs were limited to those with methoxyethane (*p* < 0.01, ϕ = 0.301, 7A middle graph). Among the drugs with both pyrimidine and methoxyethane, there were significantly more cases of ILD caused than with other drugs (*p* < 0.05, ϕ = 0.407, 7A right graph), when the drugs were limited to those binding to ABCG2.

Similar trends were observed for the drug binding to kinases (Fig. 7B). When the drugs were limited to those binding to kinases, significantly more cases of ILD caused were found among the drugs with pyrimidine (*p* < 0.05, ϕ = 0.240, 7B left graph), methoxyethane (*p* < 0.01, ϕ = 0.376, 7B middle graph), or both (*p* < 0.05, ϕ = 0.326, 7B right graph), and significantly fewer cases were found among the drugs without these substructures; there were significantly more cases of ILD causing among the drugs binding to kinases, when the drugs were limited to those with pyrimidine (*p* < 0.01, ϕ = 0.359, 7B left graph), methoxyethane (*p* < 0.01, ϕ = 0.379, 7B middle graph), or both (*p* < 0.01, ϕ = 0.539, 7B right graph), and significantly fewer cases among the drugs not binding to the kinases.

### Integration of literature information with the results of this study

Although the clarified features of drugs inducing ILD identified by the analysis so far were certainly specific to the drugs, these might merely be a part of many features and have no relation with the ILD pathogenesis. That is, although we focused on ABCG2, other targets may be involved in the induction of ILD. Moreover, given the large proportion of kinase inhibitors among all drugs that induce ILD, the features of kinase inhibitors might be similar to those of drugs that induce ILD. To test the hypothesis that inhibition of ABCG2 might induce ILD, we investigated (1) whether drugs inducing ILD at a high rate had higher inhibitory activity on ABCG2 and (2) whether drugs other than kinase inhibitors also targeted ABCG2.

Supplementary Table S10 shows the results of integrating the literature information about drugs with a high incidence of ILD (Jiang et al. 2004; Skeoch et al. 2018) and those that strongly inhibit ABCG2 (Deng et al. 2023) with the results of this study. Data from the literature are also shown in Supplementary Fig. S4. Based on these data, drugs that induce ILD can be roughly classified as follows:

1. Drugs with pyrimidine as a substructure and known to be tyrosine kinase inhibitors, which are inferred to inhibit both tyrosine kinases and ABCG2 by binding to ATP-binding sites of them. [for example, Gefitinib, Erlotinib (known targets of both: EGFR)] (N = 12)
2. Drugs known to be allosteric inhibitors of kinases (“rapalog”), which inhibit kinases by binding to sites other than the ATP binding site (Liu et al. 2023) and also shown to bind to ABCG2 (ChEMBL) and inhibit it [e.g., Temsirolimus, Everolimus (known target of both: MTOR)] (*N* = 3)
3. Drugs that are not known kinase inhibitors, but are inferred to inhibit kinases and/or ABCG2 based on ChEMBL and the literature [e.g., atorvastatin (known target: HMGCR), sulfasalazine] (*N* = 16)
4. Drugs known to be covalent inhibitors whose known and inferred targets vary. There are no data showing that these drugs bind to ABCG2 except for rabeprazole [e.g., azacitidine (known target: DNMT), carmustine (known target: DNA), clopidogrel (known target: P2RY12)] (*N* = 7)
5. Drugs other than 1)−4), which have neither information about binding to kinases nor ABCG2 (*N* = 47)

Several researchers listed drugs inducing ILD at a high rate (Jiang et al. 2004; Skeoch et al. 2018), and 15 were associated with drugs inducing ILD in this study, of which 9 drugs belonged to 1), 2), and 3), and the other 6 drugs were included in 5). Deng et al. (Deng et al. 2023) studied 232 commonly used drugs with the vesicular transport assay, and found 75 drugs that reduced the relative transport activity of ABCG2 to less than 25% of the vehicle control and were categorized as strong inhibitors. Among the above 232 drugs, 195 including 66 “strong inhibitors” corresponded to the drugs within the range of (i) and (iii) in Fig. 1 of our study. Among kinase inhibitors [1) and 2)], almost all drugs with a high incidence of ILD showed strong inhibitory activity. In contrast, among drugs other than kinase inhibitors [3), 4) and 5)], drugs inducing ILD at a high rate were divided into two groups with respect to inhibitory activity on ABCG2: there were also drugs that had little inhibitory activity on ABCG2 (Supplementary Fig. S4). The proportion of strong ABCG2-inhibitors to the drugs inducing ILD at a high rate was significantly higher than that to the drugs not inducing ILD, when the drugs were limited to those other than kinase inhibitors (*p* < 0.05) (Supplementary Table S11).

Supplementary Table S12 indicates that the protein targeted by most drugs inducing ILD was ABCG2, whereas this protein did not exist among the top 20 proteins targeted by many other drugs. When drugs were limited to those inducing ILD, the only protein targeted by many drugs common to the four drug groups [1, 2, 4, and the union of 3 and 5 based on the above classification] was ABCG2 (Supplementary Table S13).

## Discussion

DI-ILD is caused by various medications, including chemotherapeutic agents, antibiotics, antiarrhythmic drugs, and immunosuppressive agents (Skeoch et al. 2018; Yayan and Rasche 2025). Although the number of drugs associated with ILD development continues to increase, particularly with the increasing use of such drugs, the pathogenetic mechanism underlying DI-ILD remains mostly unknown (Spagnolo et al. 2022; Bridi et al. 2024; Schwaiblmair et al. 2012). This study initiated by employing data-driven approaches without setting hypotheses, yielded detailed information regarding drugs that induce ILD. Analysis of databases, from conventional statistical analysis to unsupervised machine learning, showed similarities and differences in various ILD-inducing drugs and suggested that these drugs could be roughly classified into several groups: 1) drugs that were inferred to inhibit both tyrosine kinases and ABCG2 by binding to their ATP-binding sites; 2) drugs that were inferred to inhibit kinases by binding to sites other than the ATP-binding site and to inhibit ABCG2; 3) drugs that are not known kinase inhibitors, but are inferred to inhibit kinases and/or ABCG2; 4) drugs known to be covalent inhibitors; and 5) other drugs.

ABCG2 is one of the ABC transporters which are expressed constitutively in both cancer and normal cells, participating in the process of absorption, distribution, metabolism, excretion and toxicity (Evans and McLeod 2003; Deng et al. 2014). Cancer cells promote overexpression or activation of ABC transporter, enhancing the efflux of chemotherapeutic drugs including Tyr kinase inhibitors, which decreases the intracellular drug concentrations and leads to multidrug resistance (MDR) phenotype (Wu and Fu 2018; Kathawala et al. 2015). According to recent studies, Tyr kinase inhibitors can reverse MDR by blocking the function of ABC transporter and subsequently promote drug accumulation (Ozvegy-Laczka et al. 2004). Actually, most Tyr kinase inhibitors have the ability to effectively target the ATP-binding sites of kinases and inhibit the function of ABC transporters by competing with ATP (Wang and Fu 2010; Baillache and Unciti-Broceta 2020; Roskoski 2020). Even Tyr kinase inhibitors that are not known inhibitors of ABC transporters may inhibit them because Tyr kinase inhibitors are endowed with substrate-like properties at low concentrations, whereas at high concentrations, they likely act as inhibitors of ABC transporters (Wu and Fu 2018; Brózik et al. 2011). The comparison of drug groups in our study indicated that, among Tyr kinase inhibitors, more drugs tended to bind to ABCG2 than to other drugs, confirming the results of the hierarchical clustering of targets by ligands. Moreover, the percentages of ABCG2 inhibitors in both the Ser/Thr and Tyr kinase inhibitor groups were higher than those of other drugs. In contrast, the percentage of kinase inhibitors in the ABCG2/ABCB1 inhibitor group was higher than that of the other drugs. These results are consistent with those of previous studies (Shukla et al. 2009; Eadie et al. 2014). Notably, only the drug group inducing ILD included significantly more ABCG2 inhibitors and kinase inhibitors than those in the other drug groups inducing some disease, although the drug groups including ABCG2 substrates at a higher rate also induced TEN and VAS, as well as ILD, and the drug groups including more drugs merely binding to ABCG2 induced TEN, NS, and BP, as well as ILD.

In addition to the direct phosphorylation of ABC transporters, kinases may contribute to signaling cascades that ultimately affect the transporter trafficking and/or activity (Crawford et al. 2018). The authors of the report hypothesized that ABCG2 expression is mediated by both Akt and MAPK pathways in response to epidermal growth factor (EGF) stimulation in several cell lines and under various conditions. Moreover, according to a nested case–control study by Jo et al., of the 42 categories of drugs investigated, EGFR inhibitors (OR 16.84) and class III antiarrhythmic drugs (OR 7.01) were associated with the highest disease risk of ILD (Jo et al. 2021). Taken together, these results suggest that drugs that induce ILD alter the trafficking, activity, and expression of ABCG2 by binding to kinases as well as ABCG2 itself.

The results of substructure searches for drugs containing pyrimidine and methoxyethane showed that ILD-inducing drugs contained the query substructures at a high rate. Moreover, subsequent analysis for the relationship between the substructures of a drug and its binding to targets indicated that drugs satisfying both conditions of “binding to ABCG2/kinase” and “having substructure pyrimidine/methoxyethane” were associated with ILD more than those with one condition, suggesting that these substructures are responsible for inhibiting the proteins. As mentioned previously, many kinase inhibitors mimic ATP to block its binding to kinases (Jug and Ilas 2025). As for pan-ABC transporter inhibitors, the six primary basic scaffolds of pan-ABC transporter inhibitors were identified as 4-anilinopyrimidine, pyrrolo[3,2-d]pyrimidine, pyrimido[5,4-*b*]indole, quinazoline, quinoline, and thieno[2,3-*b*]pyridine (Namasivayam et al. 2021). Kinase and ABC transporter inhibitors often contain pyrimidines as a common substructure. Similarly, the methoxy moiety is known to be favorable for EGFR inhibition (Șandor et al. 2023), and, simultaneously, it is a substructure of the ABCG2 inhibitor (Namasivayam et al. 2021, 2022; Zanden et al. 2005), although it is not known to be a basic scaffold of ABCG2. Based on these findings, it is conceivable that many drugs associated with ILD have characteristics of inhibiting ABCG2 and kinases with high affinity.

PCA indicated the following relationships: 1) among Tyr kinase inhibitors, ABCG2 inhibitor scaffolds, affinity to ABCG2 and kinases, 2) among Ser/Thr kinase inhibitors, methoxyethane, affinity to ABCG2 and kinases, and 3) covalent inhibitors. Although PC1 was strongly correlated with Tyr kinase inhibitors and ABCG2/ABCB1 inhibitors, a separation between the drugs inducing ILD and other drugs was found, but there was also an overlap between them. Subsequent comparisons among Tyr kinase inhibitors, Ser/Thr kinase inhibitors, and covalent inhibitors in PC1 and PC7 revealed differences between these groups. In this regard, drug-induced ILD is not exactly homogeneous with respect to the target, pharmacological action, or substructure.

Although the pathogenesis of DI-ILD is largely unknown for most drugs, the mechanisms by which drug-induced lung injury occurs are likely to involve direct damage to alveolar epithelial or capillary endothelial cells, dysregulation of the immune system, systemic cytokine release, cell-mediated lung damage, and free radical production with oxidative injury (Spagnolo et al. 2022; Matsuno 2012). As mentioned above, many kinase inhibitors are known to induce ILD with a high incidence rate; however, the reason for this is unclear. Moreover, the reason why drugs other than kinase inhibitors cause a common ILD phenotype remains to be clarified. Although many kinase inhibitors are promiscuous compounds, our study suggests that at least ABCG2 is involved in ILD, considering that only ABCG2 was the target common to the top 20 targets in each drug group (i.e.,Tyr kinase inhibitors, Ser/Thr inhibitors, Covalent inhibitors, and Others). The results of z-Test for the difference between two proportions showing that the proportion of strong ABCG2-inhibitors to the drugs inducing ILD at a high rate was significantly higher than that to the drugs not inducing ILD, when the drugs were limited to those other than kinase inhibitors, also supports the hypothesis. Hence, ABCG2 inhibition as distinguished from kinase inhibition is likely to be associated with ILD. If the inhibition of ABCG2 is the exact cause of DI-ILD, modification of responsible drugs may lead to clinical improvement. According to Valdameri et al., the number and position of methoxy substituents appear to be critical for both inhibition and cytotoxicity of ABCG2 (Valdameri et al. 2012). Therefore, alteration of these features of methoxy moiety of existing drugs may reduce side effects.

As ABCG2 is expressed in cells of various tissues as well as human alveolar epithelial type 2 (AT2) and type 1-like (AT1-like) cells, the question remains as to why ABCG2 inhibition affects the lung epithelium. As for Tyr kinase inhibitors, a possible explanation is that the drugs intracellularly accumulated through the inhibition of ABCG2 by the inhibitors have high cytotoxicity. It is noteworthy that both gefitinib and erlotinib, the drugs for Non-Small-Cell Lung Cancer (NSCLC) targeting EGFR, cause ILD at a high rate and are known to inhibit the transport activity of ABCG2 and reverse ABCG2-mediated drug resistance (Wu and Fu 2018; Ozvegy-Laczka et al. 2004; Yanase et al. 2004; Noguchi et al. 2009). Another possibility could come from the existence of drugs with little evidence regarding binding to ABCG2. Among drugs other than kinase inhibitors, a few are known to induce ILD at a high rate, despite little evidence that they inhibit ABCG2. One example of the drugs is methotrexate (MTX), which was originally designed as a folate pathway antagonist (Friedman and Cronstein 2019) and has long been suspected to be a causative agent of lung diseases, including fibrotic ILD (Kaplan and Waite 1978; Phillips et al. 1987; Carson et al. 1987). Similarly, pemetrexed (PEM), an anti-folate drug that exerts an antitumor effect by inhibiting folate metabolism (Adjei 2004), occasionally causes ILD (Tomii et al. 2017; Otoshi et al. 2019). Incidentally, both MTX and PEM are chemical analogs of FA and have pyrimidine as substructure. However, not all pyrimidine substructures have been implicated in ABCG2 binding. Another report showed that cytosolic FA disruption through both FA deficiency and treatment with PEM promotes metastatic propensity or contributes to EGFR-TKI (Tyr kinase inhibitor) resistance through epithelial-mesenchymal transition (EMT) induction in NSCLC cells (Shen et al. 2021). EMT is also implicated in pulmonary fibrosis pathogenesis (e.g. ILD) (Li et al. 2025). ABCG2 has known to be a folate efflux transporter, expressed in various tissues (e.g., small intestine, liver) and related to tissue folate absorption (Visentin et al. 2014; Qu et al. 2020). On the other hand, it is recently reported that this transporter is not primarily extruding its substrates out of the cell, but rather to an intracellular compartment where FAs can be kept as storage and the maintenance of cellular FA homeostasis (Ifergan et al. 2004; Lemos et al. 2008). There is also a report that loss of ABC transporter expression brings about a significant expansion in the intracellular reduced FA pool (Assaraf 2006). Moreover, sulfasalazine, an ABCG2 inhibitor inducing ILD, is known to impair folic acid absorption (Ratajczak et al. 2021). Based on these findings, the effect of ABCG2 inhibition on the amount of FA in human alveolar epithelial cells may be contrary to that on drugs transported by ABCG2. In this context, it is suggested that abnormal FA metabolism may be a common mechanism of DI-ILD. A study using the human alveolar epithelial cell line A549 showed that FA prevented MTX-induced EMT, indicating that FA may be a useful pharmaceutical for treating MTX-induced lung injury (Kawami et al. 2018). Similarly, FA may alleviate ILD caused by other drugs. Our study simultaneously shows the possibility that inhibiting FA metabolism underlies the anti-tumor effects of molecular-targeting agents, especially Tyr kinase inhibitors. This hypothesis is supported by the fact that FA metabolism is critical for cell proliferation. Better treatment methods need to be developed for preventing the side effects of ILD, considering the possibility.

There are some possible limitations in this study. First, our conclusions are mainly based on statistical associations derived from database analysis. To establish a direct causal role of ABCG2 inhibition in ILD, validation studies for the exposure and outcome are required. Such studies should consider key determinants such as dosage and tissue specificity. In this regard, the proposed ABCG2-ILD relationship is interpreted as hypothesis-generating finding rather than a confirmed mechanistic pathway. Second, database coverage is limited. Although ABCB1 is also targeted by many of the same drugs as ABCG2 and known to contribute to the phenomenon of cancer-related MDR (Wu and Fu 2018), the amount of the data of interaction between drugs and ABCB1 in the databases was not enough to analyze. There were also drugs which did not reach the range for analysis because of a deficiency of themselves in the databases used (e.g., monoclonal antibodies) or limitations in data extraction (e.g., drugs targeting no human protein), even if these are known to induce ILD. Further information on side effects and drug-target interactions in vitro and in silico are required to elucidate the molecular mechanisms of drug-induced diseases.

## Conclusions

The findings of our study revealed one of the features of drugs that induce ILD: inhibition of ABCG2 as well as kinases through the substructures of pyrimidine and methoxyethane. Additionally, considering that many strong inhibitors of ABCG2 may or may not be kinase inhibitors, among drugs reported to have a high incidence of ILD, suggests that ABCG2 inhibition is likely to be associated with ILD. Drugs without any evidence of binding to both ABCG2 and kinases despite inducing ILD at a high rate were also found, including anti-folate. Given that ABCG2 is a folate efflux transporter, abnormal FA metabolism is suspected to be a common phenomenon of DI-ILD, in addition to the anti-tumor effects of molecular-targeting agents, especially Tyr kinase inhibitors. Considering this possibility, treatments can be improved to prevent ILD as a side effect.

## Supplementary Information

Supplementary Material 1.

## Data Availability

The scripts and datasets supporting the conclusions of this article are available in the FigShare repository, https://doi.org/10.6084/m9.figshare.31771030.

## Abbreviations

ABC transporter: ATP-binding cassette (ABC) transporter
ABCG2: Broad substrate specificity ATP-binding cassette transporter ABCG2 isoform 1
ILD: Interstitial lung disease
FA: Folic acid
ABCB1: ATP-dependent translocase ABCB1 isoform 2
MAPT: Microtubule-associated protein tau isoform 1
TDP1: Tyrosyl-DNA phosphodiesterase 1 isoform a
RID: Rare and intractable disease
DI-ILD: Drug-induced interstitial lung disease
TEN: Toxic epidermal necrolysis
SLE: Systemic lupus erythematosus
NS: Nephrotic syndrome
VAS: Vasculitis
BP: Bladder pain
Ser/Thr kinase: Serine/threonine kinase
Tyr kinase: Tyrosine kinase
PCA: Principal component analysis
GPCR: G protein-coupled receptor
KCNH2: Voltage-gated inwardly rectifying potassium channel KCNH2 isoform a
HDAC6: Protein deacetylase HDAC6 isoform b
ADRB1: Beta-1 adrenergic receptor
ADRB2: Beta-2 adrenergic receptor
ATP: Adenosine triphosphate
EGFR: Epidermal growth factor receptor isoform a precursor
MTOR: Serine/threonine-protein kinase mTOR isoform 1
HMGCR: 3-Hydroxy-3-methylglutaryl-Coenzyme A reductase isoform 1
DNMT: DNA (cytosine-5)-methyltransferase 1 isoform b
DNA: (Deoxyribonucleotide)n
P2RY12: P2Y purinoceptor 12
Akt: RAC-alpha serine/threonine-protein kinase
MDR: Multidrug resistance
MAPK: Mitogen-activated protein kinase 1
EGF: Epidermal growth factor
NSCLC: Non-Small-Cell Lung Cancer
EMT: Epithelial-mesenchymal transition
MTX: Methotrexate
PEM: Pemetrexed
