# Supplementary material for: Drugs inducing interstitial lung disease: in silico analysis of their structures and targets
Source: Mol Med. 2026 Jul 21;32:190. doi: 10.1186/s10020-026-01559-5 (PMC13678984; doi:10.1186/s10020-026-01559-5)
Supplement: Supplementary file 1 [file 10020_2026_1559_MOESM1_ESM.pdf]

Supplementary Information

Drugs inducing interstitial lung disease: *in silico* analysis of their structures and targets

Mao Tanabe, Shokichi Takahama, Takuya Yamamoto, Ryuichi Sakate

Center for Intractable Diseases and ImmunoGenomics (CiDIG), National Institutes of Biomedical Innovation, Health and Nutrition (NIBN), Ibaraki, Osaka 567-0085, Japan.

CONTENTS	Page No
Table S1: ABCG2 substrates/inhibitors and ABCB1 substrates/inhibitors.	3-6
Table S2: SMILES used for substructure search in this study: Basic scaffolds of ABC transporter (ABCG2, ABCB1, ABCC1) inhibitors (PMID:37714963, 33724808).	7
Table S3: SMILES used for substructure search in this study: Methoxyethane.	7
Table S4: SMILES used for substructure search in this study: warheads oriented towards cysteine (Current research in Chemical Biology 3 (2023)100040 Serafim.R.A.M., et al).	8-9
Table S5: SMILES used for substructure search in this study: warheads oriented towards Cys, Lys, Catalytic Ser/Thr, Asp/Glu, and Met/His/Ser (PMID: 33275063 (Fig.4)).	10-15
Table S6: Top 10 proteins registered as targets of many drugs in ChEMBL and DrugBank.	16
Table S7: The diseases caused as side effects by many drugs binding to one of four proteins (i.e., ABCG2, ABCB1, TDP1, MAPT）	17
Table S8: Example of Chi-square test	17
Figure S1: Distribution of the percentage of drugs targeting a protein to each of the drug groups.	18
Figure S2: Distribution of the percentage of drugs of a category to each of the drug groups.	19-20
Figure S3: Distribution of the percentage of drugs containing a substructure to each of the drug groups.	21
Table S9: Principal component analysis (PCA)	22-23
Table S10: Integration of literature information to the results of this study (Drugs inducing ILD only)	24-25
Figure S4: A dispersion chart of inhibitory activity of drugs in each of four groups on ABCG2-mediated rosuvastatin transport	26
Table S11: Proportion of “strong ABCG2 inhibitors” to the drugs classified from the points of view of whether these are kinase inhibitors and the inducibility of ILD.	27
Table S12: Comparison between the top 20 proteins targeted by drugs inducing ILD and other drugs.	28
Table S13: Comparison among the top 20 proteins targeted by drugs of four groups inducing ILD.	29

Supplementary Table S1. ABCG2 substrates/inhibitors, ABCB1substrates/inhibitors and covalent inhibitors

**ABCG2 substrates**		Ref. 23, 24, 25		
Mitoxantrone	Etoposide	Doxorubicin	Daunorubicin	Epirubicin
Topotecan	Irinotecan	SN-38	5-fluorouracil	Methotrexate
Cladribine	Clofarabine	6-mercaptopurine	Flavopiridol	Imatinib
Dasatinib	Nilotinib	Sorafenib	Sunitinib	Gefitinib
Erlotinib	Rucaparib	Pheophorbide a	Chlorin e6	HPPH
5-aminolevulinic acid	Porfimer	Fluorouracil	Ritonavir	Bisantrene
Tamoxifen	Prazosin	Nitrofurantoin	Prasterone	Estrone-3-sulfate
Folic acid	Alvocidib	Allopurinol	Teniposide	Idarubicin
**ABCB1 substrates**		Ref. 24, 25		
Mitoxantrone	Doxorubicin	Methotrexate	Nilotinib	Gefitinib
Ritonavir	Bisantrene	Tamoxifen	Prazosine	Nitrofurantoin
Paclitaxel	Cyclosporine A	Dexamethasone	Rifamycin	Verapamil
Vincristine	Ibuprofen	Phenobarbital	Atrvastatin	Saquinavir
Etoposide	Daunorubicin	Topotecan	Imatinib	Quinidine
Celiprolol	Vinblastine	Indinavir	Nelfinavir	Epirubicin
Idarubicin	Teniposide	Adriamycin	Docetaxel	Flavopyridol
Lapatinib	Mitomycin	Actinomycin	Navelbine	Irinotecan
Fluorouracil	Cyclosporin A	Mitomycin C	Actinomycin D	Asimadoline
chlorambucil	cytarabine	hydroxyurea	cefoperazone	ceftriaxone
clarithromycin	doxycycline	erythromycin	gramicidin A	gramicidin D
grepafloxacin	itraconazole	ketoconazole	posaconazole	levofloxacin
rifampicin	sparfloxacin	tetracycline	valinomycin	cimetidine
fexofenadine	terfenadine	azidopine	bepridil	dilitazem
felodipine	nifedipine	nisoldipine	nitrendipine	tiapamil
lopinavir	amprenavir	maraviroc	chlorpromazine	phenothiazine
trifluoperazine	loperamide	amiodarone	lidocaine	propafenone
cyclosporin H	sirolimus	tacrolimus	everolimus	hydrocortisone

corticoserone	triamcinolone	phenytoin	topiramate	oxcarbazepine
carbamazepine-10,11-epoxide	eslicarbazepine acetate	saxagliptin	sitagliptin	aliskiren
ambrisentan	talinolol	tolvaptan	epidril	calcein acetoxymethyl ester
Hoechst 33342	rhodamine 123	BCECFAM	calcein AM	endosulfan
leupeptin	methyl parathion	paraquat	pepstatin A	transflupentixol
ivermectin	abamectin	emetine	reserpine	aristolochic acid
dabigartan etexilate	ranolazine	digoxin	lovastatin	simvastatin
colchicine	domperidone	ondansetron	curcuminoid	flavonoid
**ABCG2 inhibitors**		Ref. 24, 25, 26		
Ko143	Fumitremorgin C	Elacridar	Tariquidar	Nilotinib
Quercetin	NSC73306	Flavoperidol	Chrysin	Benzoflavone
Estradiol	Novobiocin	Vismodegib	Sulfasalazine	Febuxostat
DHW-221	Abemaciclib	Ribociclib	Alisertib	IPI-549
WDR5-0103	SKLB610	Erlotinib	Midostaurin	Lazertinib
Anlotinib	OSI-027	CC-671	BEZ235	MY-5445
Adagrasib	Imatinib	Nirotinib	Dasatinib	Enasidenib
Tepotinib	Venetoclax	Ketoconazole	Lapatinib	Infigratinib
Sonidegib	Erdafitinib	Entrectinib	Sitravatinib	TMP195
Dronedarone	Crizotinib	Almonertinib	Dacomitinib	Saracatinib
Pantoprazole	esomeprazole	Diltiazem	Noscapine derivatives	Febuxostat
Everolimus	Veliparib	Gefitinib	Sunitinib	Vandetanib
**ABCB1 inhibitors**		Ref. 24, 25, 27, 28		
Elacridar	DHW-221	Abemaciclib	Ribociclib	Alisertib
IPI-549	WDR5-0103	SKLB610	Erlotinib	Erlotinib
Lazertinib	Anlotinib	Yrazolo[3,4-d] pyrimidine scaffod	Celastrol andits derivatives	OSI-027

CC-671	BEZ235	MY-5445	Adagrasib	Imatinib
Nirotinib	Dasatinib	Enasidenib	Tepotinib	Venetoclax
Ketoconazole	Lapatinib	Infigratinib	Sonidegib	Erdafitinib
Entrectinib	Sitravatinib	TMP195	Dronedarone	Crizotinib
Almonertinib	Dacomitinib	Saracatinib	Pantoprazole	esomeprazole
Diltiazem	Noscapine derivatives	Febuxostat	Everolimus	Veliparib
Gefitinib	Sunitinib	Vandetanib	verapamil	Cyclosporin A
CP100356	CBT-1	zosuquidar	dofequidar	valspodar
laniquidar	S 9788	biricodar	XR9051	tariquidar
vincristine	reserpine	quinidine	tamoxifen	trifluoperazine
dexniguldipine	bricodar	ONT-093	mitotane	annamycin
**Covalent inhibitors**		Ref. 29, 30		
Acetylsalicylic acid	Propylthiouracil	Mechlorethamine	Disulfiram	Penicillin G
Isoniazid	Phenoxybenzamine	Warfarin	Busulfan	Penicillin V
Chlorambucil	Thiotepa	Cyclophosphamide	Ampicillin	Fluorouracil
D-cycloserine	Melphalan	Floxuridine	Amoxicillin	Carbidopa
Dacarbazine	Lomustine	Carmustine	Cisplatin	Cefaclor
Trifluridine	Estramustine	Streptozocin	Ceftriaxone	Clavulanic acid
Cephalexin	Sulbactam	Ifosfamide	Cefuroxime axetil	Omeprazole
Selegiline	Carboplatin	Eflornithine	Altretamine	Cefprozil
Finasteride	Tazobactam	Lansoprazole	Gemcitabine	Fosfomycin
Meropenem	Clopidogrel	Cefdinir	Capecitabine	Rabeprazole
Exemestane	Temozolomide	Orlistat	Pantoprazole	Rivastigmine
Esomeprazole	Dutasteride	Oxaliplatin	Bortezomib	Azacitidine
Decitabine	Rasagiline	Bendamustine	Vigabatrin	Saxagliptin
Ceftaroline fosamil	Abiraterone	Boceprevir	Telaprevir	Carfilzomib
Ibrutinib	Afatinib	Dimethyl fumarate	Ceftolozane	Osimertinib
Trabectedin	Avibactam	Ixazomib	Acalabrutinib	Neratinib

Vaborbactam	Dacomitinib	Zanubrutinib	Selinexor	Voxelotor
Cefiderocol	Relebactam			

Reused from ref. 23, 29 (Creative Commons Attribution 4.0 International License, http://creativecommons.org/licenses/by/4.0/)

Reused from ref. 24 and 28 with permission of the publisher (Springer Nature)

Reused from ref. 25 (the publisher: Bentham Science)

Reused from ref. 26 with permission of the publisher (Elsevier, http://www.elsevier.com)

Reused from ref. 27 with permission of the publisher (Taylor & Francis Ltd, http://www.tandfonline.com)

Modified from ref. 30 (Table S1) with permission from the publisher (Taylor & Francis Ltd, https://www.tandfonline.com).

Supplementary Table S2: SMILES used for substructure search in this study: Basic scaffolds of ABC transporter (ABCG2, ABCB1, ABCC1) inhibitors (Ref.24, 31)

	Structure	SMILES
ABC_1	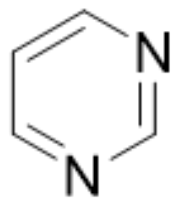	C1=CN=CN=C1<br>(Pyrimidine)
ABC_2	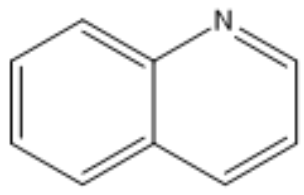	C12=CC=CC=C1N=CC=C2<br>(quinoline)
ABC_3	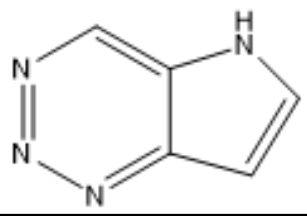	C12=CN=NN=C1C=CN2<br>(pyrrolotriazine)
ABC_4	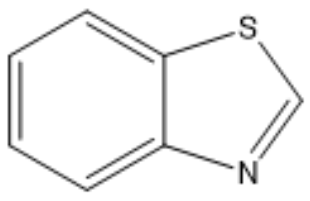	C12=CC=CC=C1SC=N2<br>(benzothiazole)
ABC_5	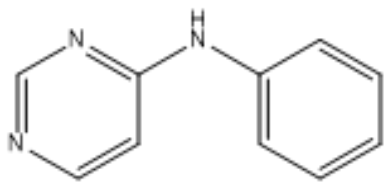	C1(NC2=CC=CC=C2)=NC=NC=C1<br>(4-anilinopyrimidine)
ABC_6	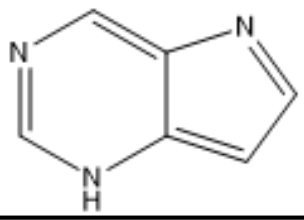	C12=CC=NC1=CN=CN2<br>(pyrrolo[3,2-d]pyrimidine)
ABC_7	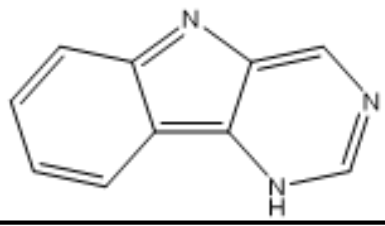	C12=CN=CNC1=C3C=CC=CC3=N2<br>(pyrimido[5,4-b]indole)
ABC_8	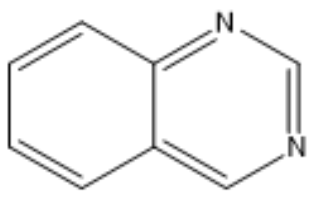	C12=CN=CN=C1C=CC=C2<br>(quinazoline)
ABC_9	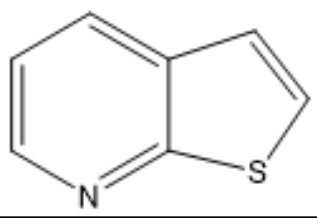	C12=NC=CC=C1C=CS2<br>(Thieno(2,3-b)pyrimidine)

Reused from ref. 24 with permission of the publisher (Springer Nature)
Reused from ref. 31 (Creative Commons Attribution 4.0 International License, http://creativecommons.org/licenses/by/4.0/)

Supplementary Table S3. SMILES used for substructure search in this study: Methoxyethane

	Structure	SMILES
9	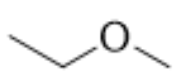	CC(OC)<br>(Methoxyethane)

Supplementary Table S4: SMILES used for substructure search in this study: warheads oriented towards cysteine (Ref. 29)

	Structural formula of warhead	SMILES
WH_Cys_1	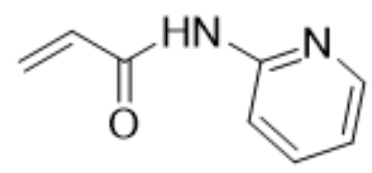	O=C(C=C)NC1=NC=CC=C1
WH_Cys_2	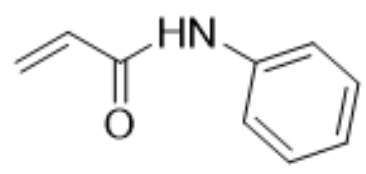	O=C(C=C)NC1=CC=CC=C1
WH_Cys_3	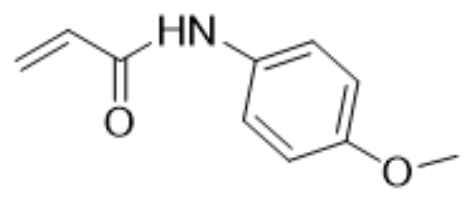	O=C(C=C)NC1=CC=C(OC)C=C1
WH_Cys_4	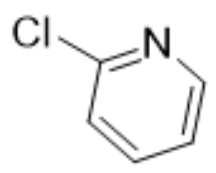	ClC1=NC=CC=C1
WH_Cys_5	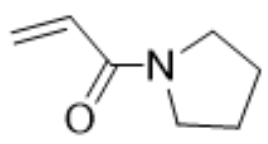	O=C(C=C)N1CCCCC1
WH_Cys_6	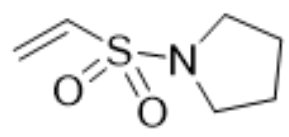	O=S(N1CCCC1)(C=C)=O
WH_Cys_7	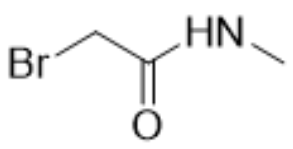	CNC(CBr)=O
WH_Cys_8	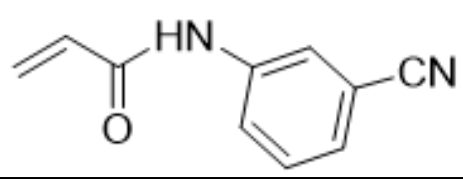	O=C(C=C)NC1=CC(C#N)=CC=C1
WH_Cys_9	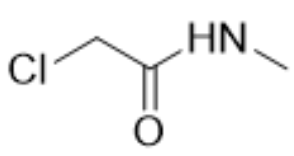	CNC(CCl)=O
WH_Cys_10	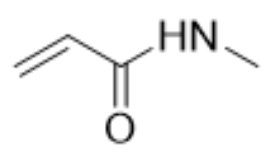	CNC(C=C)=O
WH_Cys_11	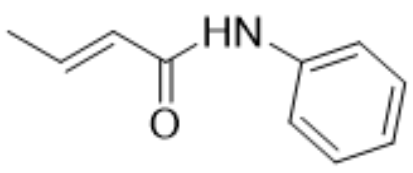	O=C(/C=C/C)NC1=CC=CC=C1
WH_Cys_12	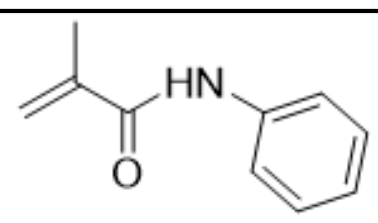	O=C(C(C)=C)NC1=CC=CC=C1
WH_Cys_13	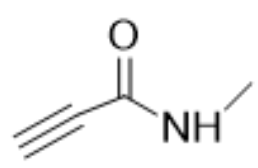	O=C(C#C)NC

Supplementary Table S4 (Continued)

	Structural formula of warhead	SMILES
WH_Cys_14	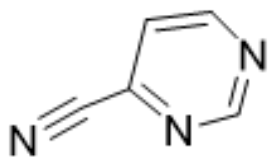	N#CC1=NC=NC=C1
WH_Cys_15	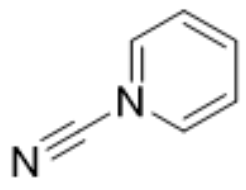	N#CN1CCCCC1
WH_Cys_16	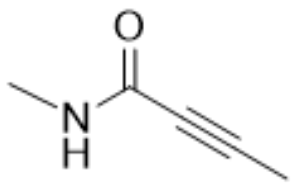	O=C(NC)C#CC
WH_Cys_17	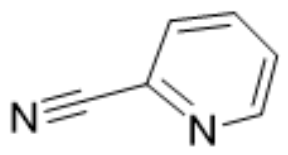	N#CC1=NC=CC=C1

Reused from ref. 29 (Creative Commons Attribution 4.0 International License, http://creativecommons.org/licenses/by/4.0/)

Supplementary Table S5: SMILES used for substructure search in this study warheads oriented towards Cys, Lys, Catalytic Ser/Thr, Asp/Glu, and Met/His/Ser (Ref.30)

	Structural formula and SMILES of warhead	ID	Structural formula	SMILES
I	C=CC(N(C)C)=O 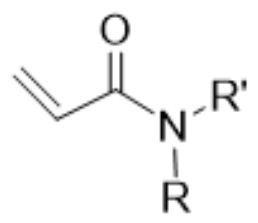 FDA approved (Cys)	WH_1_1	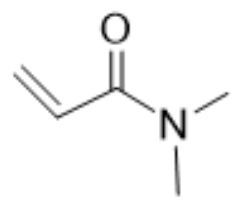	C=CC(N(C)C)=O
		WH_1_2	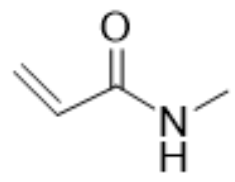	C=CC(NC)=O
II	O=C(C#C[R])N([R'])[R] 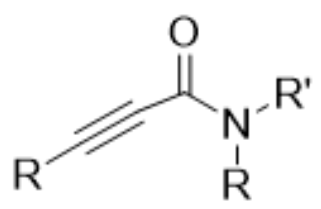 FDA approved (Cys)	WH_2_1	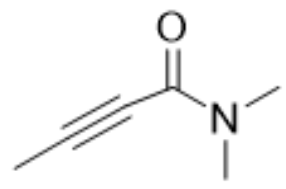	O=C(C#CC)N(C)C
		WH_2_2	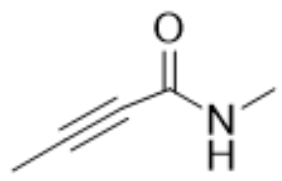	O=C(C#CC)NC
		WH_2_3	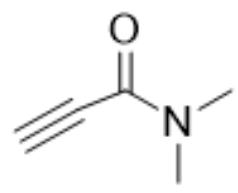	O=C(C#C)N(C)C
		WH_2_4	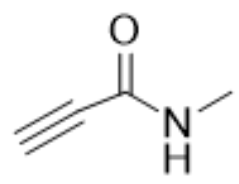	O=C(C#C)NC

Supplementary Table S5. (Continued)

	Structural formula and SMILES of warhead	ID	Structural formula	SMILES
III	O=C(/C=C/C(O[R'])=O)[X][R]<br>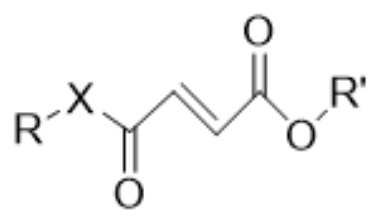<br>FDA approved (Cys)	WH_3_1	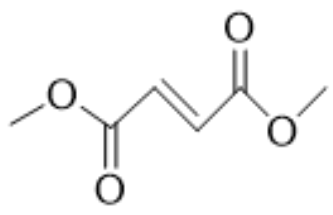	O=C(/C=C/C(OC)=O)OC
		WH_3_2	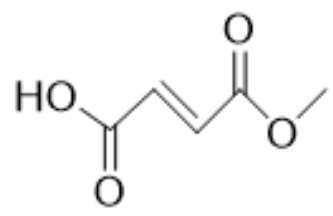	OC(/C=C/C(OC)=O)=O
		WH_3_3	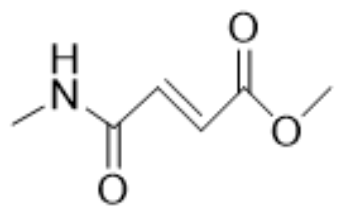	O=C(/C=C/C(OC)=O)NC
		WH_3_4	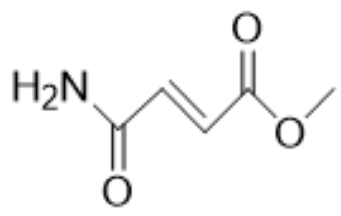	O=C(N)/C=C/C(OC)=O
		WH_3_5	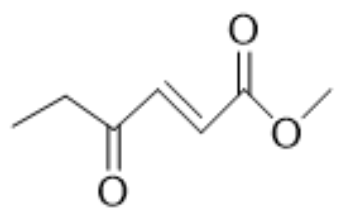	O=C(/C=C/C(OC)=O)CC
		WH_3_6	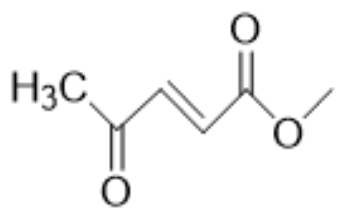	O=C(C)/C=C/C(OC)=O

Supplementary Table S5. (Continued)

	**Structural formula and SMILES of warhead**	**ID**	**Structural formula**	**SMILES**
IV	O=S([X]([R'])[R])(C=C)=O 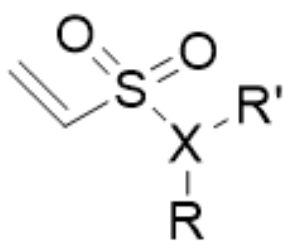	WH_4_1	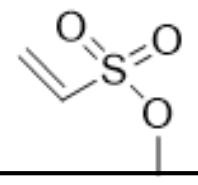	O=S(OC)(C=C)=O
		WH_4_2	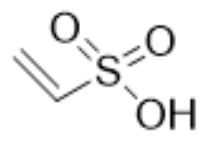	O=S(O)(C=C)=O
		WH_4_3	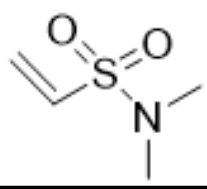	O=S(N(C)C)(C=C)=O
		WH_4_4	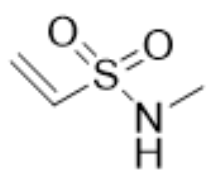	O=S(NC)(C=C)=O
		WH_4_5	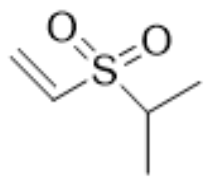	O=S(C(C)C)(C=C)=O
		WH_4_6	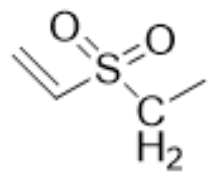	O=S(CC)(C=C)=O
V	O=C(N([R])[R'])C(F)=C 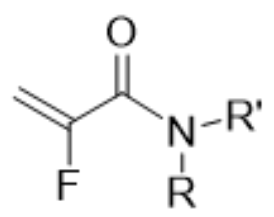	WH_5_1	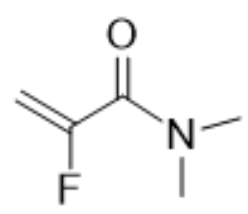	O=C(N(C)C)C(F)=C
		WH_5_2	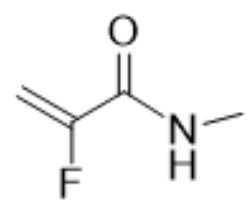	O=C(NC)C(F)=C
VI	O=C(N([R])[R'])C(C#N)=C 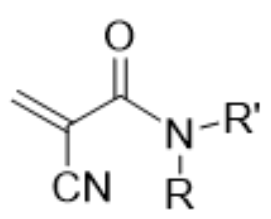	WH_6_1	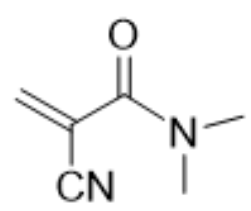	O=C(N(C)C)C(C#N)=C
		WH_6_2	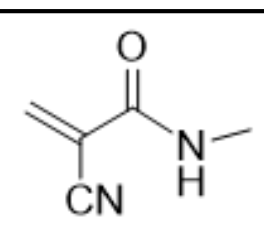	O=C(NC)C(C#N)=C
VII	N#CN([R'])[R''] 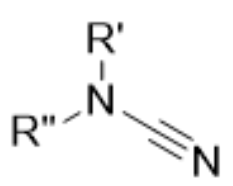	WH_7	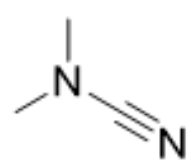	N#CN(C)C

Supplementary Table S5. (Continued)

	Structural formula and SMILES of warhead	ID	Structural formula	SMILES
VIII	[R']C#C 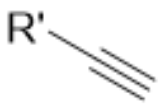	WH_8	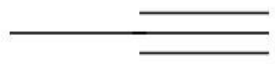	CC#C
IX	O=C(N([R])[R'])C(C)Cl 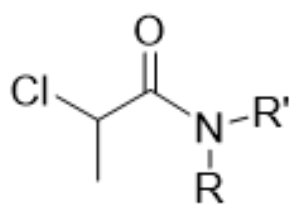	WH_9_1	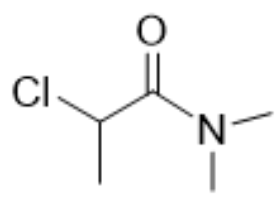	O=C(N(C)C)C(C)Cl
		WH_9_2	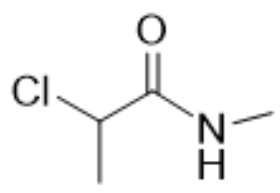	O=C(NC)C(C)Cl
X	O=C(N([R])[R'])C(Cl)F 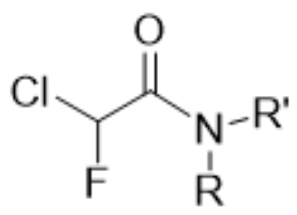	WH_10_1	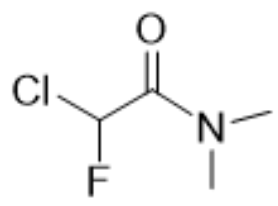	O=C(N(C)C)C(Cl)F
		WH_10_2	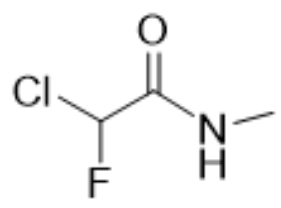	O=C(NC)C(Cl)F
XI	CC1(C)C2=CC=CC=C2N=C(C#CC)O1 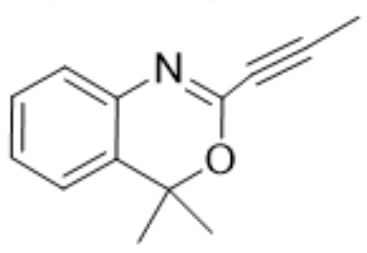	WH_11	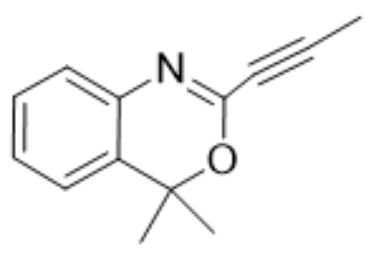	CC1(C)C2=CC=CC=C2N=C(C#CC)O1
XII	O=C1N([R'])C([R''])([R])C1 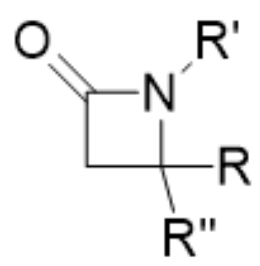 FDA approved (Catalytic Ser/Thr)	WH_12_1	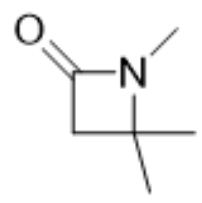	O=C1N(C)C(C)(C)C1
		WH_12_2	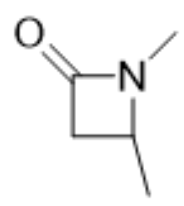	O=C1N(C)C(C)C1

Supplementary Table S5. (Continued)

	Structural formula and SMILES of warhead	ID	Structural formula	SMILES
XIII	O=C(O[R'’])[X]([R])[R’]<br>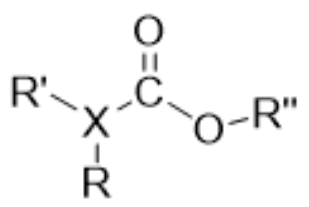<br>FDA approved (Catalytic Ser/Thr)	WH_13_1	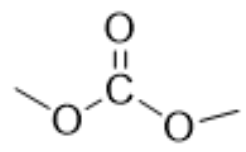	O=C(OC)OC
		WH_13_2	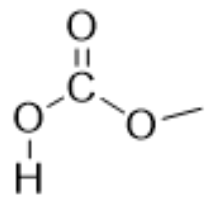	OC(OC)=O
		WH_13_3	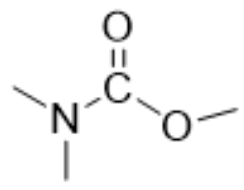	O=C(OC)N(C)C
		WH_13_4	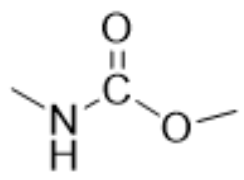	O=C(OC)NC
		WH_13_5	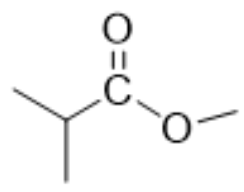	O=C(OC)C(C)C
		WH_13_6	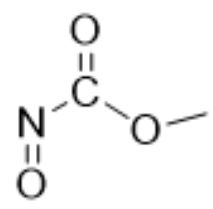	O=C(OC)N=O
XIV	[R']B(O)O 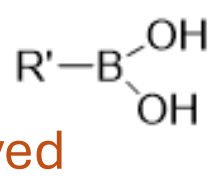<br>FDA approved (Catalytic Ser/Thr)	WH_14	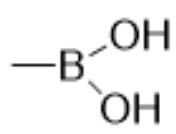	CB(O)O
XV	O=S([X][R'])(F)=O<br>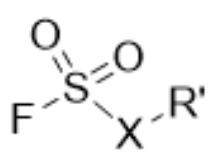	WH_15_1	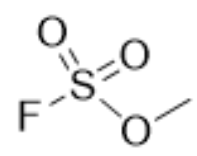	O=S(OC)(F)=O
		WH_15_2	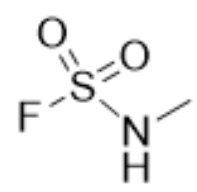	O=S(NC)(F)=O
		WH_15_3	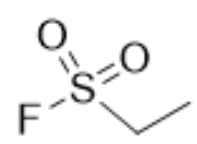	O=S(CC)(F)=O

Supplementary Table S5 (Continued)

	Structural formula and SMILES of warhead	ID	Structural formula	SMILES
XVII	C[N+]1=CC([R'])=C([R])O1 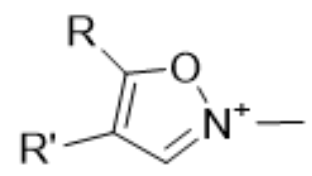	WH_17_1	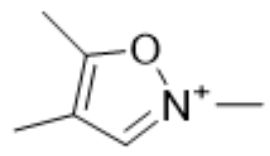	C[N+]1=CC(C)=C(C)O1
		WH_17_2	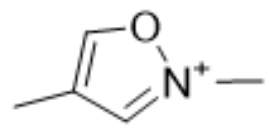	C[N+]1=CC(C)=CO1
XVIII	[R']C1C[X]1 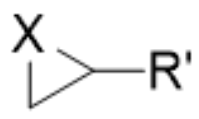	WH_18_1	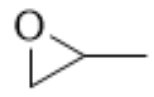	CC1CO1
		WH_18_2	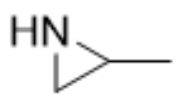	CC1CN1
		WH_18_3	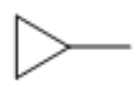	CC1CC1

Modified from ref. 30 (Fig. 4) with permission from the publisher (Taylor & Francis Ltd, https://www.tandfonline.com).

Supplementary Table S6. Comparison between the top ten proteins registered as targets of many drugs in ChEMBL and DrugBank.

**ChEMBL**	N=2566				**DrugBank**	N=2510		
UniProt ID	Gene name	SUM	(%)		UniProt ID	Gene name	SUM	(%)
Q12809	KCNH2	148	5.8		P14416	DRD2	115	4.6
P36888	FLT3	111	4.3		P35367	HRH1	107	4.3
P07949	RET	108	4.2		P11229	CHRM1	90	3.6
Q9NUW8	TDP1	104	4.1		P28223	HTR2A	84	3.3
P10636	MAPT	102	4.0		P35348	ADRA1A	79	3.1
P00533	EGFR	96	3.7		P35354	PTGS2	78	3.1
P35968	KDR	94	3.7		P08908	HTR1A	76	3.0
P14416	DRD2	92	3.6		P08172	CHRM2	74	2.9
P00519	ABL1	85	3.3		P23219	PTGS1	71	2.8
P09619	PDGFRB	79	3.1		P20309	CHRM3	68	2.7

The range of data for analysis is the union of (i), (ii), (iii), and (iv) in Fig. 1A.

Supplementary Table S7. The diseases caused as side effects by many drugs binding to one of the four proteins (i.e., ABCG2, ABCB1, TDP1, MAPT) (The diseases related to rare and intractable diseases in Japan are listed).

		Related rare and intractable disease			Target protein				Chi_square test	
					ABCG2(Total N: 33)		Other (Total N: 949)		P value	Phi (ϕ)
Rank	Side effect	ID	Disease name	Disease group	Drugs (N)	Drugs (%)	Drugs (N)	Drugs (%)		coefficient
103	Interstitial lung disease	R85	Idiopathic interstitial pneumonia	Resp	15	45.5	71	7.5	**	0.242
127	Toxic epidermal necrolysis	R39	Toxic epidermal necrolysis	Skin	14	42.4	188	19.8	**	0.101
167	Bladder pain	R226	Interstitial cystitis with Hunners ulcer	Kid	11	33.3	141	14.9	**	0.092
310	Vasculitis	R42	Polyarteritis nodosa	Imm	6	18.2	118	12.4	NS	
328	Nephrotic syndrome	R222	Primary nephrotic syndrome	Kid	5	15.2	40	4.2	**	0.094

		Related rare and intractable disease			Target protein				Chi_square test	
					ABCB1(Total N: 16)		Other (Total N: 966)		P value	Phi (ϕ)
Rank	Side effect	ID	Disease name	Disease group	Drugs (N)	Drugs (%)	Drugs (N)	Drugs (%)		coefficient
223	Toxic epidermal necrolysis	R39	Toxic epidermal necrolysis	Skin	5	31.3	197	20.4	NS	
264	Interstitial lung disease	R85	Idiopathic interstitial pneumonia	Resp	4	25.0	82	8.5	*	0.074

		Related rare and intractable disease			Target protein				Chi_square test	
					MAPT(Total N: 32)		Other (Total N: 950)		P value	Phi (ϕ)
Rank	Side effect	ID	Disease name	Disease group	Drugs (N)	Drugs (%)	Drugs (N)	Drugs (%)		coefficient
108	Toxic epidermal necrolysis	R39	Toxic epidermal necrolysis	Skin	10	31.3	192	20.2	NS	
228	Vasculitis	R42	Polyarteritis nodosa	Imm	5	15.6	119	12.5	NS	
296	Bladder pain	R226	Interstitial cystitis with Hunners ulcer	Kid	4	12.5	148	15.6	NS	

		Related rare and intractable disease			Target protein				Chi_square test	
					TDP1(Total N: 35)		Other (Total N: 947)		P value	Phi (ϕ)
Rank	Side effect	ID	Disease name	Disease group	Drugs (N)	Drugs (%)	Drugs (N)	Drugs (%)		coefficient
77	Systemic lupus erythematosus	R49	Systemic lupus erythematosus	Imm	10	28.6	58	6.1	**	0.164
88	Vasculitis	R42	Polyarteritis nodosa	Imm	9	25.7	115	12.1	*	0.076
148	Toxic epidermal necrolysis	R49	Toxic epidermal necrolysis	Skin	6	17.1	196	20.7	NS	

The range of data for analysis is the union of (i) and (iii) in Fig. 1A. Chi-Square tests with Holm correction for multiple comparisons were done to calculate *p* values [the number of multiple comparisons is 5 (Target: ABCG2), 2 (Target: ABCB1) or 3 (Target: MAPT, TDP1); * $p<0.05$, ** $p<0.01$].

Supplementary Table S8. Example of Chi-square test.

Rank: 103 (Side effect) Interstitial lung disease

Actual

	Binding_to_ABCG2	Not_binding_to_ABCG2
Causing the SE	15	71
Not causing the SE	18	878

Expected

	Binding_to_ABCG2	Not_binding_to_ABCG2
Causing the SE	2.9	83.1
Not causing the SE	30.1	865.9

Chi-square test (p)
3.29732E-14

Supplementary Figure S1

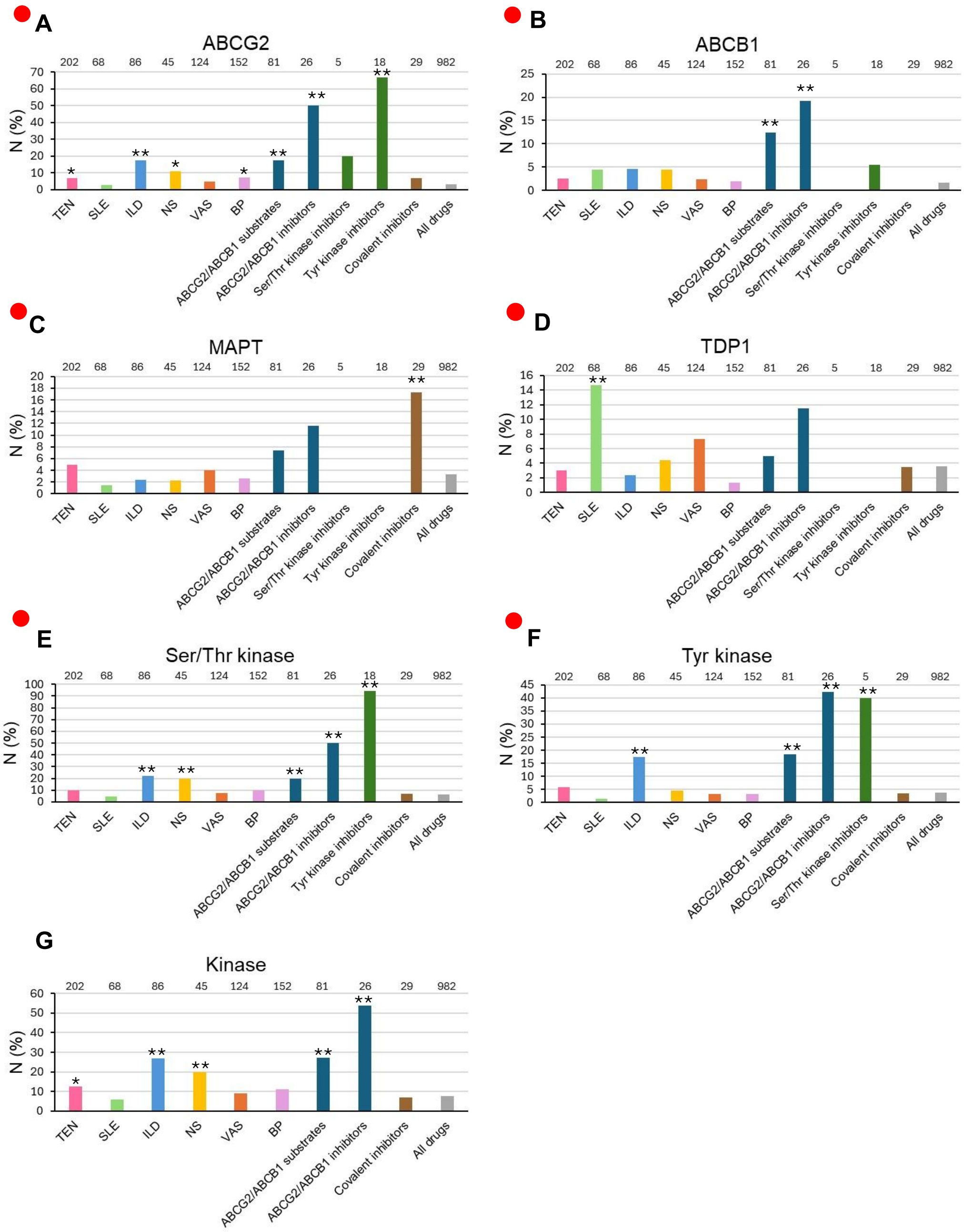

Supplementary Fig. S1. Distribution of the percentage of drugs targeting a protein to each of the drug groups. Each protein is shown as gene name: ABCG2 (A), ABCB1(B), MAPT (C), TDP1 (D), Ser/Thr kinase (E), Tyr kinase (F), or (Ser/Thr or Tyr) kinase (G). The upper number of each bar is the total number of drugs. Chi-Square tests with Holm correction for multiple comparisons were done to calculate *p* values [the number of multiple comparisons is 11 (A, B, C, D), 10 (E, F), or 9(G); * $p<0.05$, ** $p<0.01$].The range of data for analysis is (i) and (iii) in Fig. 1A. Bar graphs marked with red circles are also shown in Fig.3.

Supplementary Figure S2

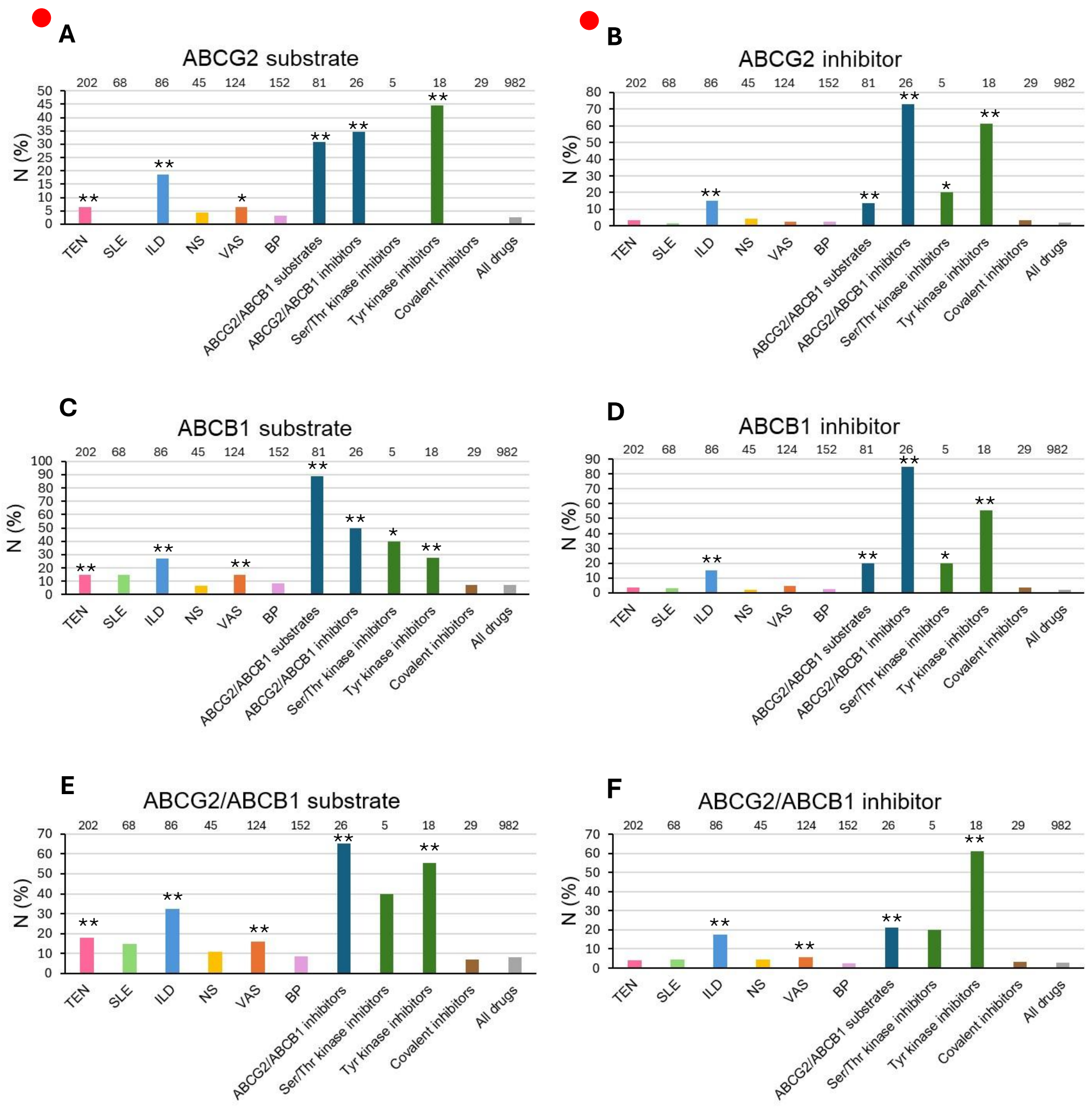

Supplementary Figure S2 (continued)

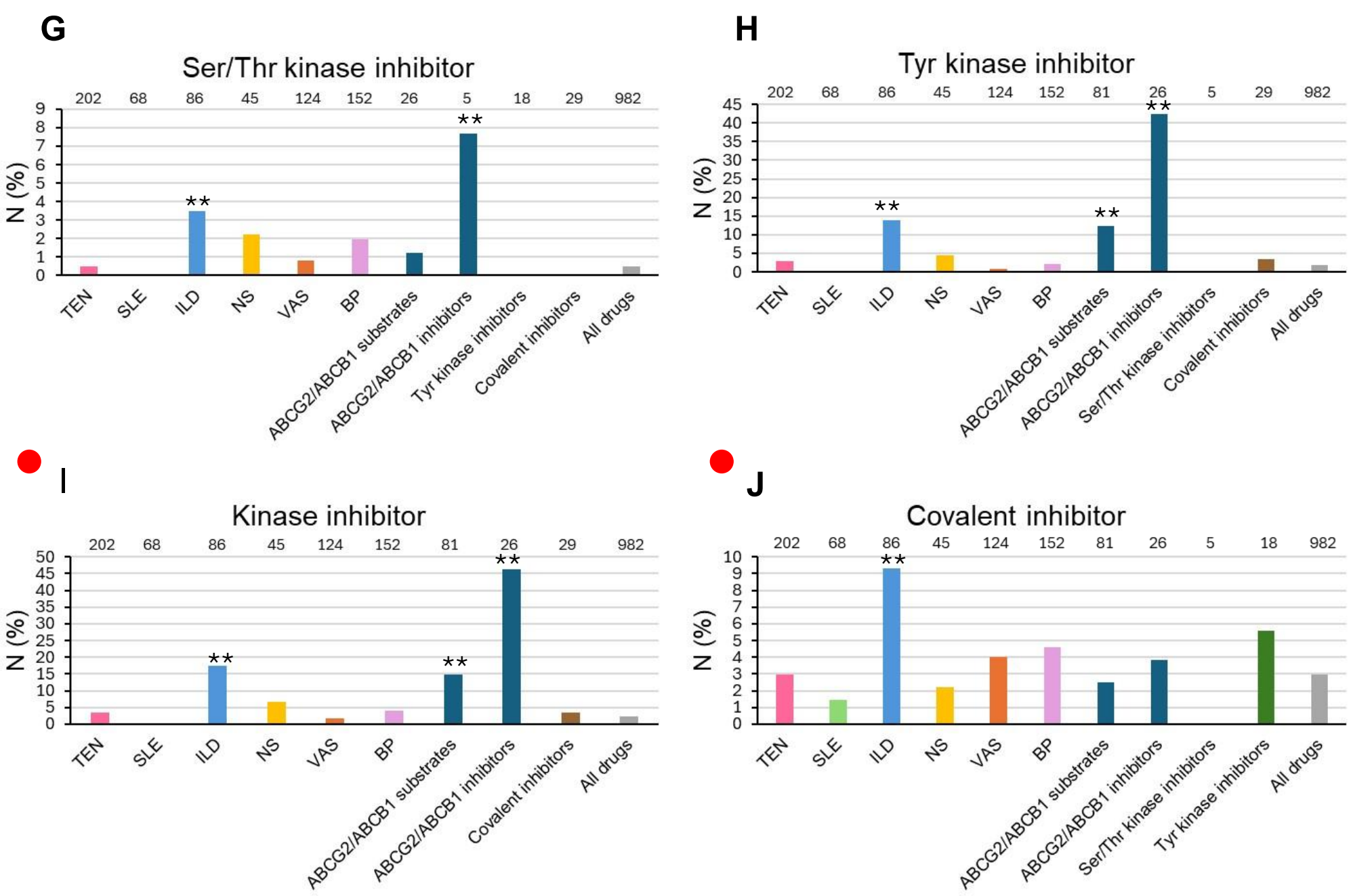

Supplementary Fig.S2. Distribution of the percentage of drugs of a category to each of the drug groups. The categories are ABCG2 substrate (A), ABCG2 inhibitor (B), ABCB1 substrate (C), ABCB1 inhibitor (D), ABCG2/ABCB1 substrate (E), ABCG2/ABCB1 inhibitor (F), Ser/Thr kinase inhibitor (G), Tyr kinase inhibitor (H), (Ser/Thr or Tyr) kinase inhibitor (I), and covalent inhibitor (G). The upper number of each bar is the total number of drugs. The upper number of each bar is the total number of drugs. Chi-Square tests with Holm correction for multiple comparisons were done to calculate *p* values [the number of multiple comparisons is 11 (A, B, C, D), 10 (E, F, G, H, J), 9 (I); * $p<0.05$, ** $p<0.01$].The range of data for analysis is (i) and (iii) in Fig. 1A. Bar graphs marked with red circles are also shown in Fig.4.

Supplementary Figure S3

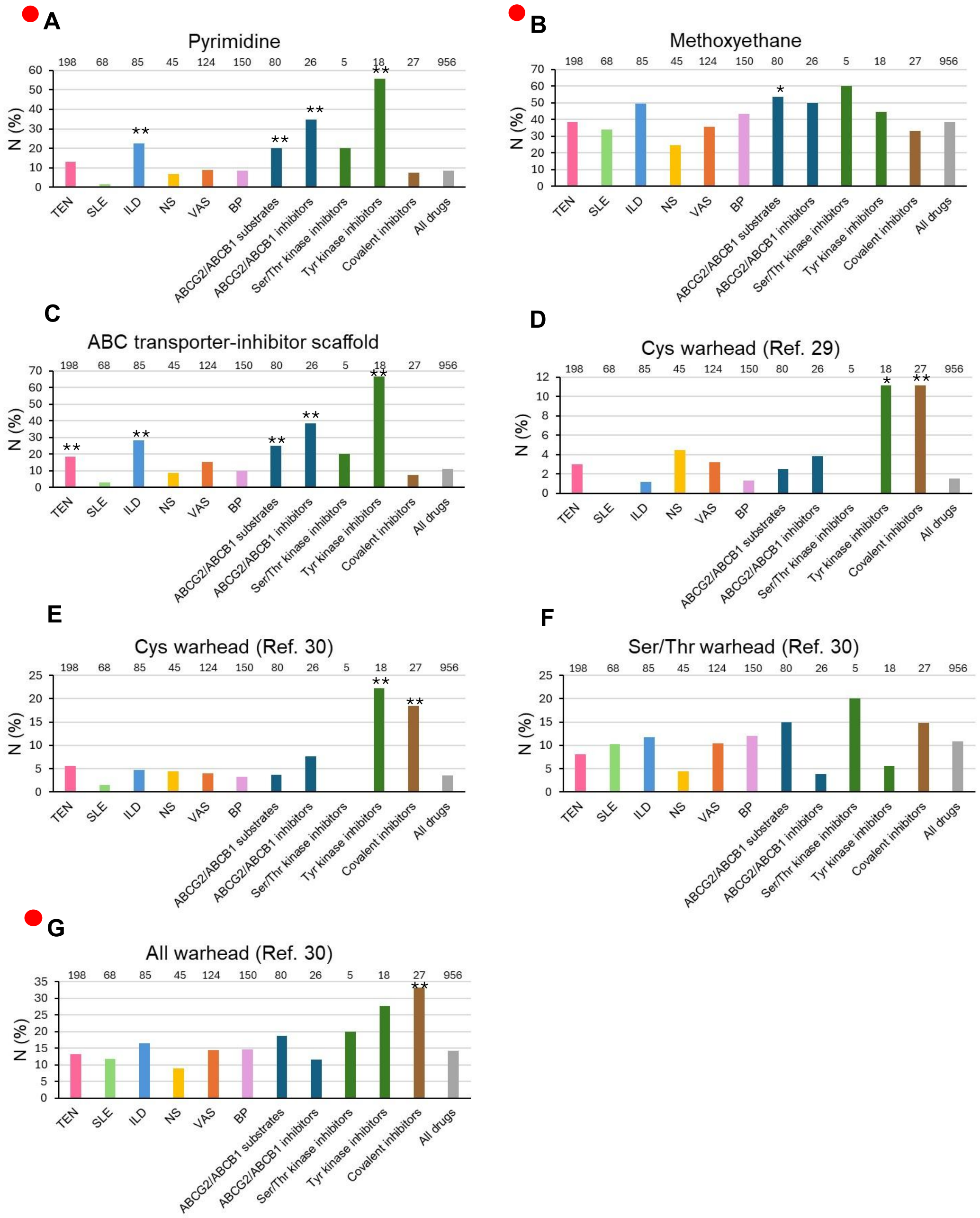

Supplementary Fig.S3. Distribution of the percentage of drugs containing a substructure to each of the drug groups. The substructures are pyrimidine (A), methoxyethane (B), ABC transporter-inhibitor scaffold (C), Cys warhead (Ref. 29) (D), Cys warhead (Ref. 30) (E), Ser/Thr warhead (Ref. 30) (F), or all warheads of covalent inhibitor (Ref. 30) (G). The upper number of each bar is the total number of drugs. The upper number of each bar is the total number of drugs. Chi-Square tests with Holm correction for multiple comparisons were done to calculate *p* values [the number of multiple comparisons is 11; * $p<0.05$, ** $p<0.01$]. The range of data for analysis is (i) in Fig. 1A. Bar graphs marked with red circles are also shown in Fig.5.

Supplementary Table S9: The correlations between the principal components and the original valuables

	PC1	PC2	PC3	PC4	PC5	PC6	PC7	PC8	PC9	PC10
ABCG2_substrate	0.442841	-0.17787	0.019012	-0.01577	-0.00808	-0.20786	0.251796	-0.15197	-0.08434	-0.01703
ABCB1_substrate	0.290802	-0.14127	0.004877	-0.03116	-0.03347	0.028296	0.395593	-0.35526	-0.12405	-0.04437
ABCG2_inhibitor	0.666017	-0.26922	0.059085	-0.0051	0.017709	-0.18483	0.071867	-0.14655	-0.08032	0.060331
ABCB1_inhibitor	0.60927	-0.25235	0.051126	-0.01258	0.009088	-0.19443	0.163672	-0.2473	-0.11785	0.054093
Ser_Thr_kinase_inhibitor	0.174905	-0.12299	0.090424	-0.01099	-0.01926	0.679762	0.056551	-0.04561	0.096369	0.021121
Tyr_kinase_inhibitor	0.777108	-0.22493	0.014204	0.009178	0.024988	-0.17617	-0.09424	0.092144	0.018436	-0.00182
Covalent_inhibitor	0.066868	0.098417	-0.06463	0.003818	-0.01552	-0.05866	-0.01095	0.080438	-0.00761	0.219629
ABCG2	0.543222	-0.23826	0.063448	-0.0017	0.004351	-0.04473	0.058917	-0.1335	-0.15316	0.037263
ABCB1	0.16741	-0.12101	0.041063	-0.0214	-0.01719	-0.03188	0.286442	-0.33222	-0.20552	0.011672
MAPT	0.001255	-0.0333	-0.00162	-0.01073	-0.03264	-0.0964	0.266575	-0.12886	-0.44878	0.020771
TDP1	-0.02299	-0.02622	-0.00051	-0.00829	-0.0305	-0.07166	0.198983	-0.20654	-0.37692	-0.01941
MTOR	0.162259	-0.11955	0.088999	-0.01578	-0.02309	0.70948	0.097565	-0.08552	0.080576	0.019064
STK	0.654344	-0.25547	0.096465	0.00976	-0.00461	0.453463	-0.10862	0.185072	-0.02989	-0.01203
TK	0.704993	-0.25229	0.053191	0.014017	0.014234	0.03831	-0.13411	0.169537	-0.00594	-0.03699
Kinase	0.680486	-0.27973	0.10323	0.010085	-0.00483	0.431608	-0.10508	0.191422	-0.03927	-0.02439
Methoxyethane	0.042289	-0.02531	-0.09191	-0.1238	-0.0006	0.008432	0.417968	-0.19479	0.462096	0.04837
ABC_1_Pyrimidine	0.353951	-0.17059	0.02181	0.008904	0.005912	-0.24574	-0.14379	0.160833	0.20726	-0.03019
ABC_2_quinoline	0.032131	-0.04627	0.020708	0.005057	-0.02111	0.11534	0.019148	0.054622	-0.07854	-0.0951
ABC_3_pyrrolotriazine										
ABC_4_benzothiazole	-0.01255	0.002149	-0.00035	0.004132	-0.00136	-0.00296	-0.03353	0.006059	-0.02505	-0.01614
ABC_5_4_anilinopyrimidine	0.540386	-0.12336	-0.0316	0.003684	0.025978	-0.30371	-0.10648	0.110578	0.241198	0.029317
ABC_6_pyrrolo_pyrimidine										
ABC_7_pyrimido_indole										
ABC_8_quinazoline	0.457135	-0.12987	-0.02277	0.002083	0.023325	-0.37782	-0.10771	0.09804	0.303752	0.02356
ABC_9_Thieno_pyrimidine										
WH_Cys_1	0.05841	0.222166	-0.01658	0.007479	-0.0047	0.03529	-0.0626	0.034392	-0.14337	0.809112
WH_Cys_2	0.466257	0.659202	-0.44908	0.003653	-0.00266	0.034592	0.02872	-0.01755	0.024819	-0.26281
WH_Cys_3										
WH_Cys_4										
WH_Cys_5										
WH_Cys_6										
WH_Cys_7										
WH_Cys_8										
WH_Cys_9	-0.01681	0.003347	-0.00137	0.711278	0.694066	0.023075	0.102869	-0.00866	0.019384	0.006838
WH_Cys_10	0.394554	0.803467	-0.02838	0.002145	-0.00363	0.045898	-0.01493	0.019899	-0.06162	0.324015
WH_Cys_11	0.466257	0.659202	-0.44908	0.003653	-0.00266	0.034592	0.02872	-0.01755	0.024819	-0.26281
WH_Cys_12	0.215762	0.516304	-0.44652	-0.0022	-0.01107	0.088538	0.083771	-0.08747	0.003968	-0.31252
WH_Cys_13										
WH_Cys_14										
WH_Cys_15										
WH_Cys_16										
WH_Cys_17										
WH_1_1	0.158145	0.572306	0.525915	-0.00937	0.005687	-0.02487	0.032793	-0.01106	0.016558	-0.09721
WH_1_2	0.394554	0.803467	-0.02838	0.002145	-0.00363	0.045898	-0.01493	0.019899	-0.06162	0.324015
WH_2_1										
WH_2_2										
WH_2_3										
WH_2_4										
WH_3_1										
WH_3_2	-0.01184	0.001546	-0.00489	-0.00405	-0.0014	-0.00233	0.003308	-0.01595	0.045357	-0.00534

Supplementary Table S9 (Continued)

	PC1	PC2	PC3	PC4	PC5	PC6	PC7	PC8	PC9	PC10
WH_3_3										
WH_3_4										
WH_3_5										
WH_3_6										
WH_4_1										
WH_4_2										
WH_4_3										
WH_4_4										
WH_4_5										
WH_4_6										
WH_5_1										
WH_5_2										
WH_6_1	0.086312	0.428917	0.835791	-0.01774	0.011056	-0.06764	0.058445	-0.01915	0.061235	-0.16144
WH_6_2	0.086312	0.428917	0.835791	-0.01774	0.011056	-0.06764	0.058445	-0.01915	0.061235	-0.16144
WH_7										
WH_8	0.073627	-0.05227	0.007605	0.009717	-0.0011	-0.1241	-0.1134	0.120747	0.122342	0.045855
WH_9_1										
WH_9_2										
WH_9_3										
WH_9_4	-0.01681	0.003347	-0.00137	0.711278	0.694066	0.023075	0.102869	-0.00866	0.019384	0.006838
WH_10_1										
WH_10_2										
WH_11										
WH_12_1										
WH_12_2	-0.01255	0.002149	-0.00035	0.004132	-0.00136	-0.00296	-0.03353	0.006059	-0.02505	-0.01614
WH_13_1	-0.01586	0.002408	-0.01908	-0.69685	0.714468	0.018152	-0.01015	0.022791	-0.0351	0.002261
WH_13_2	-0.01586	0.002408	-0.01908	-0.69685	0.714468	0.018152	-0.01015	0.022791	-0.0351	0.002261
WH_13_3	-0.02834	-0.01172	-0.01857	-0.03118	-0.05231	-0.03278	0.6199	0.643242	-0.04017	0.019973
WH_13_4	-0.03828	-0.01762	-0.02288	-0.03784	-0.05682	-0.04402	0.655347	0.607819	-0.01409	0.01047
WH_13_5	-0.03588	-0.02495	-0.01999	-0.05013	-0.0351	0.082187	0.361648	-0.32688	0.513376	0.143311
WH_13_6										
WH_14	-0.01255	0.002149	-0.00035	0.004132	-0.00136	-0.00296	-0.03353	0.006059	-0.02505	-0.01614
WH_15_1										
WH_15_2										
WH_15_3										
WH_17_1										
WH_17_2										
WH_18_1	-0.03291	0.004079	-0.02104	-0.02536	-0.01536	0.016454	0.150754	-0.19863	0.473937	0.186788
WH_18_2										
WH_18_3	-0.03442	-0.00618	-0.01146	-0.003	-0.01565	0.005421	-0.03083	0.034931	0.068486	-0.04434

The range of data for analysis is (i) in Fig. 1A.

Supplementary Table S10. Integration of literature information to the results of this study (Drugs inducing ILD only).

			High incidence A (Ref. 31) B (Ref. 32)		Strong inhibition of ABCG2 (Ref. 33, Supplementary Table 2)	Known pharmacological action				Target			Substructure	
ChEMBL ID	DrugBank ID	SIDER_name	A	B	Relative transport at 50μM (%)	ABCG2 inhibitor	Ser/Thr kinase inhibitor	Tyr kinase inhibitor	Covalent inhibitor	ABCG2	STK	TK	Methoxyethane	Pyrimidine
CHEMBL1173655	DB08916	afatinib	0	0	1.8	0	0	1	1	0	1	1	1	1
CHEMBL1336	DB00398	sorafenib	0	0	-0.4	0	0	1	0	1	1	1	0	0
CHEMBL1421	DB01254	dasatinib	0	0	6.1	1	0	1	0	0	1	1	0	1
CHEMBL24828	DB05294	vandetanib	0	0	49.8	1	0	1	0	1	1	1	1	1
CHEMBL255863	DB04868	nilotinib	0	0	2.4	1	0	1	0	1	1	1	0	1
CHEMBL553	DB00530	erlotinib	1	1	3.2	1	0	1	0	1	1	1	1	1
CHEMBL601719	DB08865	crizotinib	0	0	42.5	1	0	1	0	0	1	1	1	0
CHEMBL941	DB00619	imatinib	0	0	3.2	1	0	1	0	1	1	1	0	1
CHEMBL554	DB01259	lapatinib	0	0	1.1	1	0	1	0	1	1	1	1	1
CHEMBL939	DB00317	gefitinib	0	1	2.7	1	0	1	0	1	1	1	1	1
CHEMBL5416410		dasatinib	0	0	6.1	1	0	1	0	1	1	1	0	1
CHEMBL1201179		lapatinib	0	0	1.1	1	0	1	0	0	0	1	1	1
CHEMBL413	DB00877	rapamycin	0	1		0	1	0	0	1	1	0	1	0
CHEMBL1201182	DB06287	temsirolimus	*1	1	1.3	0	1	0	0	0	1	0	1	0
CHEMBL1908360	DB01590	everolimus	1	1	2.2	1	1	0	0	0	1	0	1	0
CHEMBL421	DB00795	sulfasalazine	0	0	1	1	0	0	0	1	1	0	0	0
CHEMBL1487	DB01076	atorvastatin	1		1.1	0	0	0	0	1	0	0	0	0
CHEMBL806	DB00499	flutamide	0	0	10.7	0	0	0	0	0	0	0	0	0
CHEMBL1017	DB00966	telmisartan	0	0	14.5	0	0	0	0	0	0	0	0	0
CHEMBL269732	DB00864	tacrolimus	0	0	3.6	0	0	0	0	1	1	0	1	0
CHEMBL960	DB01097	leflunomide	1	1	9.7	0	0	0	0	0	0	1	0	0
CHEMBL633	DB01118	amiodarone	1	1	23.8	0	0	0	0	0	0	0	1	0
CHEMBL428647	DB01229	paclitaxel	1	0	12.7	0	0	0	0	0	0	1	1	0
CHEMBL1201753	DB08860	pitavastatin	0	0	2.8	0	0	0	0	0	0	0	0	0
DB00188	DB00188	bortezomib	0	0	17.5	0	0	0	0	0	0	0	0	0
CHEMBL184412	DB04855	dronedarone	0	0		1	0	0	0	0	0	0	1	0
CHEMBL58	DB01204	mitoxantrone	0	0	82.3	0	0	0	0	1	0	1	0	0
CHEMBL1397	DB01263	posaconazole	0	0	30.8	0	0	0	0	1	0	0	1	0
CHEMBL83	DB00675	tamoxifen	0	0	26.2	0	0	0	0	0	1	0	1	0
CHEMBL848	DB00480	lenalidomide	0	0	93.5	0	0	0	0	0	1	0	0	0
DB00995	DB00995	auranofin	0	0		0	0	0	0	0	1	0	1	0
CHEMBL1518	DB00550	propylthiouracil	0	0		0	0	0	1	0	0	0	0	1
CHEMBL887	DB01367	rasagiline	0	0		0	0	0	1	0	0	0	0	0
CHEMBL1771	DB00758	clopidogrel	0	0	33	0	0	0	1	0	0	0	1	0
CHEMBL1489	DB00928	5-azacytidine	0	0	98.4	0	0	0	1	0	0	0	1	0
CHEMBL1219	DB01129	rabeprazole	0	0		0	0	0	1	1	0	0	1	0
CHEMBL480	DB00448	lansoprazole	0	0	52.3	0	0	0	1	0	0	0	1	0
CHEMBL513	DB00262	bcnu	0	0		0	0	0	1	0	0	0	0	0

The range of data for analysis is (i) in Fig. 1A.

Supplementary Table S10 (Continued)

			High incidence A (Ref. 31) B (Ref. 32)		Strong inhibition of ABCG2 (Ref. 33, Supplementary Table 2)	Known pharmacological action				Target			Substructure	
ChEMBL ID	DrugBank ID	SIDER_name	A	B	Relative transport at 50 μM (%)	ABCG2 inhibitor	Ser/Thr kinase inhibitor	Tyr kinase inhibitor	Covalent inhibitor	ABCG2	STK	TK	Methoxyethane	Pyrimidine
CHEMBL1619	DB00242	cladribine	0	0	89.3	0	0	0	0	0	0	0	1	1
CHEMBL803	DB00987	cytarabine	0	0		0	0	0	0	0	0	0	1	1
DB00441	DB00441	gemcitabine	1	1	98.6	0	0	0	0	0	0	0	1	1
DB00290	DB00290	bleomycin	1	1	100.4	0	0	0	0	0	0	0	1	1
CHEMBL225072	DB00642	pemetrexed	0	1		0	0	0	0	0	0	0	0	1
CHEMBL34259	DB00563	methotrexate	1	1	81.5	0	0	0	0	0	0	0	0	1
CHEMBL2360464		pemetrexed	0	0		0	0	0	0	0	0	0	0	1
DB00993	DB00993	azathioprine	0	0	96.4	0	0	0	0	0	0	0	0	1
CHEMBL55	DB00738	pentamidine	0	0		0	0	0	0	0	0	0	1	0
CHEMBL1064	DB00641	simvastatin	0	0	1.8	0	0	0	0	0	0	0	1	0
CHEMBL1144	DB00175	pravastatin	0	0	80.9	0	0	0	0	0	0	0	1	0
CHEMBL723	DB01136	carvedilol	0	0	28.4	0	0	0	0	0	0	0	1	0
CHEMBL84	DB01030	topotecan	0	0	32.6	0	0	0	0	0	0	0	1	0
CHEMBL44657	DB00773	etoposide	0	0	80.1	0	0	0	0	0	0	0	1	0
CHEMBL866	DB01024	mycophenolate	0	0	89.6	0	0	0	0	0	0	0	1	0
CHEMBL1643	DB00811	ribavirin	0	0	78.7	0	0	0	0	0	0	0	1	0
CHEMBL814	DB00176	fluvoxamine	0	0	107.5	0	0	0	0	0	0	0	1	0
CHEMBL652	DB01195	flecainide	0	0		0	0	0	0	0	0	0	1	0
CHEMBL799	DB01166	cilostazol	0	0		0	0	0	0	0	0	0	1	0
CHEMBL163	DB00503	ritonavir	0	0	7.2	0	0	0	0	0	0	0	1	0
CHEMBL92	DB01248	docetaxel	1	0	31.7	0	0	0	0	0	0	0	1	0
CHEMBL786		tamoxifen	0	0	26.2	0	0	0	0	0	0	0	1	0
CHEMBL3989887		tacrolimus	0	0	3.6	0	0	0	0	0	0	0	1	0
DB01165	DB01165	ofloxacin	0	0		0	0	0	0	0	0	0	1	0
DB08871	DB08871	eribulin	*1	0		0	0	0	0	0	0	0	1	0
CHEMBL902	DB00927	famotidine	0	0	112.8	0	0	0	0	0	0	0	0	0
CHEMBL589	DB00268	ropinirole	0	0		0	0	0	0	0	0	0	0	0
CHEMBL1274	DB00665	nilutamide	0	0		0	0	0	0	0	0	0	0	0
CHEMBL409	DB01128	bicalutamide	*1	0	14.7	0	0	0	0	0	0	0	0	0
CHEMBL834	DB00282	pamidronate	0	0		0	0	0	0	0	0	0	0	0
CHEMBL21731	DB00934	maprotiline	0	0		0	0	0	0	0	0	0	0	0
CHEMBL1118	DB06700	desvenlafaxine	0	0		0	0	0	0	0	0	0	0	0
CHEMBL637	DB00285	venlafaxine	0	0	98.5	0	0	0	0	0	0	0	0	0
CHEMBL43452	DB08910	pomalidomide	0	0	94.7	0	0	0	0	0	0	0	0	0
CHEMBL1138	DB00973	ezetimibe	0	0		0	0	0	0	0	0	0	0	0
CHEMBL1479	DB01406	danazol	0	0		0	0	0	0	0	0	0	0	0
CHEMBL425	DB01250	olsalazine	0	0		0	0	0	0	0	0	0	0	0
CHEMBL572	DB00698	nitrofurantoin	1	0		0	0	0	0	0	0	0	0	0
CHEMBL468	DB01041	thalidomide	0	0	92.1	0	0	0	0	0	0	0	0	0
CHEMBL1201066		venlafaxine	0	0	98.5	0	0	0	0	0	0	0	0	0
DB01208	DB01208	sparfloxacin	0	0		0	0	0	0	0	0	0	0	0
DB00535	DB00535	cefdinir	0	0		0	0	0	0	0	0	0	0	0
DB00261	DB00261	anagrelide	0	0		0	0	0	0	0	0	0	0	0
DB00461	DB00461	nabumetone	0	0		0	0	0	0	0	0	0	0	0
DB00666	DB00666	nafarelin	0	0		0	0	0	0	0	0	0	0	0
DB00740	DB00740	riluzole	*1	0		0	0	0	0	0	0	0	0	0
DB00776	DB00776	oxcarbazepine	0	0	93.5	0	0	0	0	0	0	0	0	0

The range of data for analysis is (i) in Fig. 1A.

Supplementary Figure S4

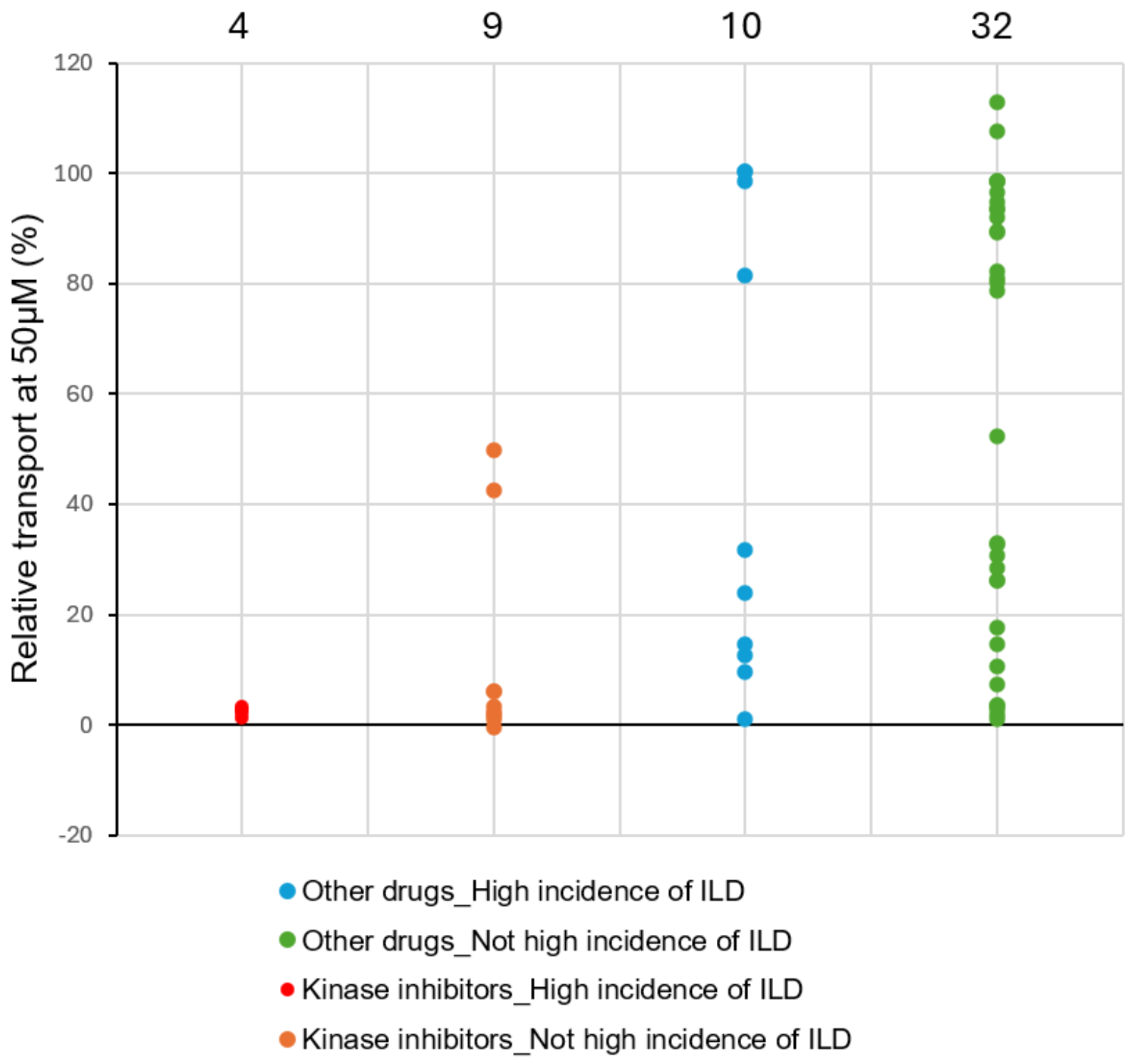

Supplementary Fig. S4. A dispersion chart of inhibitory activity of drugs in each of four groups on ABCG2-mediated rosuvastatin transport. The groups are: kinase inhibitors associated with a high incidence of ILD, kinase inhibitors not to be associated with a high incidence of ILD, other drugs associated with a high incidence of ILD, and those not to be associated with a high incidence of ILD. The incidences of ILD are reported in Ref. 32, and Ref. 33. Inhibitory activity on ABCG2-mediated rosuvastatin transport is indicated by relative transport: the transport of Lucifer yellow (LY) in the presence of 50 μM of investigated drug relative to the transport in the vehicle (DMSO) control (Ref. 34, Supplementary Table S10). The upper number of the dots with each color is the total number of drugs.

Supplementary Table S11. Proportion of “strong ABCG2 inhibitors” to the drugs classified from the points of view of whether these are kinase inhibitors and the inducibility of ILD.

		Drugs with a high incidence of ILD	Drugs inducing ILD (not high rate)	Drugs not inducing ILD
Kinase inhibitors	Sample size	4	9	7
	Strong ABCG2 inhibitors (%)	100	77.80	100
Drugs other than kinase inhibitors	Sample size	10	32	133
	Strong ABCG2 inhibitors (%)	50.00	28.13	25.56

Only the drugs corresponding to those studied in ref. 34 and lying within the union of (i) and (iii) in Fig. 1 were analyzed. The proportion of “strong ABCG2 inhibitors” to the drugs inducing ILD at a high rate is significantly higher than that to the drugs not inducing ILD, when the drugs are limited to those other than kinase inhibitors.

Supplementary Table S12. Comparison between the top 20 proteins targeted by drugs inducing ILD and other drugs. Numbers in each row indicate that of drugs targeting the protein and the percentage of it. The range of data for analysis is the union of (i) and (iii) in Fig. 1A.

Targets of drugs inducing ILD (N=86)			
UniProt_ID	Gene_name	(SUM)	(%)
Q9UNQ0	ABCG2	15	17.4
P00533	EGFR	11	12.8
P04626	ERBB2	9	10.5
P42685	FRK	9	10.5
P07948	LYN	9	10.5
P00519	ABL1	9	10.5
P06239	LCK	9	10.5
P10721	KIT	8	9.3
P42684	ABL2	8	9.3
O94804	STK10	7	8.1
P29322	EPHA8	7	8.1
Q9H2G2	SLK	7	8.1
Q08345	DDR1	7	8.1
Q12809	KCNH2	7	8.1
P11274	BCR	7	8.1
P09619	PDGFRB	7	8.1
P07949	RET	7	8.1
P36888	FLT3	7	8.1
O14976	GAK	6	7.0
P16234	PDGFRA	6	7.0

Targets of other drugs (N=896)			
UniProt_ID	Gene_name	(SUM)	(%)
P14416	DRD2	77	8.6
P35348	ADRA1A	71	7.9
Q12809	KCNH2	71	7.9
P35367	HRH1	68	7.6
P28223	HTR2A	66	7.4
P11229	CHRM1	64	7.1
P08913	ADRA2A	63	7.0
P08908	HTR1A	59	6.6
P08172	CHRM2	55	6.1
P35368	ADRA1B	55	6.1
P28335	HTR2C	55	6.1
P31645	SLC6A4	54	6.0
P20309	CHRM3	52	5.8
P23975	SLC6A2	50	5.6
P35462	DRD3	50	5.6
P34969	HTR7	49	5.5
P08588	ADRB1	46	5.1
P07550	ADRB2	45	5.0
P18089	ADRA2B	45	5.0
P18825	ADRA2C	45	5.0

Supplementary Table S13. Comparison among the top 20 proteins targeted by drugs of four groups inducing ILD. In each row, number of drugs targeting the protein and the percentage of it are indicated.

Targets of drugs inducing ILD _Tyr kinase inhibitors (N=12)			
UniProt_ID	Gene_name	(SUM)	(%)
P00533	EGFR	11	91.7
P04626	ERBB2	9	75.0
P42685	FRK	9	75.0
P07948	LYN	9	75.0
P00519	ABL1	9	75.0
P06239	LCK	9	75.0
P10721	KIT	8	66.7
Q9UNQ0	ABCG2	8	66.7
P42684	ABL2	8	66.7
O94804	STK10	7	58.3
P29322	EPHA8	7	58.3
Q9H2G2	SLK	7	58.3
Q08345	DDR1	7	58.3
P11274	BCR	7	58.3
P09619	PDGFRB	7	58.3
P07949	RET	7	58.3
P36888	FLT3	7	58.3
O14976	GAK	6	50.0
P16234	PDGFRA	6	50.0
Q16832	DDR2	6	50.0

Targets of drugs inducing ILD _Ser/Thr kinase inhibitors (N=3)			
UniProt_ID	Gene_name	(SUM)	(%)
P42345	MTOR	3	100.0
P14618	PKM	1	33.3
Q13451	FKBP5	1	33.3
P62942	FKBP1A	1	33.3
P68106	FKBP1B	1	33.3
Q9UNQ0	ABCG2	1	33.3
Q02790	FKBP4	1	33.3
Q14576	ELAVL3	0	0.0
P08579	SNRPB2	0	0.0
P32239	CCKBR	0	0.0
P54577	YARS1	0	0.0
O00418	EEF2K	0	0.0
Q96DP5	MTFMT	0	0.0
Q8TD43	TRPM4	0	0.0
P01563	IFNA2	0	0.0
P41595	HTR2B	0	0.0
P21127	CDK11B	0	0.0
P35580	MYH10	0	0.0
P02774	GC	0	0.0
P41143	OPRD1	0	0.0

Targets of drugs inducing ILD _Covalent inhibitors (N=7)			
UniProt_	Gene_nan	(SUM)	(%)
P20648	ATP4A	2	28.6
P49327	FASN	2	28.6
P10636	MAPT	2	28.6
P27338	MAOB	1	14.3
P21397	MAOA	1	14.3
P22079	LPO	1	14.3
P22303	ACHE	1	14.3
P05164	MPO	1	14.3
Q9H244	P2RY12	1	14.3
P06276	BCHE	1	14.3
Q8TCT1	PHOSPHC	1	14.3
P49895	DIO1	1	14.3
Q6SZW1	SARM1	1	14.3
Q8NFI3	ENGASE	1	14.3
P50406	HTR6	1	14.3
Q9UNQ(	ABCG2	1	14.3
P10415	BCL2	1	14.3
P26358	DNMT1	1	14.3
P07202	TPO	1	14.3
P00390	GSR	1	14.3

Targets of drugs inducing ILD _Others (N=64)			
UniProt_ID	Gene_nai	(SUM)	(%)
Q12809	KCNH2	6	9.4
Q96FL8	SLC47A1	5	7.8
Q9UNQ0	ABCG2	5	7.8
P35354	PTGS2	4	6.3
P31645	SLC6A4	4	6.3
P04035	HMGCR	4	6.3
P04818	TYMS	4	6.3
P23975	SLC6A2	4	6.3
Q86VL8	SLC47A2	4	6.3
P10275	AR	4	6.3
P11388	TOP2A	4	6.3
Q92769	HDAC2	3	4.7
P08183	ABCB1	3	4.7
Q96SW2	CRBN	3	4.7
Q01959	SLC6A3	3	4.7
P01375	TNF	3	4.7
P35869	AHR	3	4.7
P23219	PTGS1	3	4.7
Q14524	SCN5A	3	4.7
Q9UKT9	IKZF3	3	4.7
